# A Review of Plant-Mediated ZnO Nanoparticles for Photodegradation and Antibacterial Applications

**DOI:** 10.3390/nano14141182

**Published:** 2024-07-11

**Authors:** Dorcas Mutukwa, Raymond Tichaona Taziwa, Lindiwe Khotseng

**Affiliations:** 1 Department of Chemistry, University of the Western Cape, Robert Sobukwe Rd., Private Bag X17, Bellville 7535, South Africa; 4266762@myuwc.ac.za; 2 Department of Applied Science, Faculty of Science Engineering and Technology, Walter Sisulu University, Old King William Town Road, Potsdam Site, East London 5200, South Africa

**Keywords:** plant extracts, biosynthesis, green synthesis, photocatalysis, dye treatment, wastewater treatment, antibacterial agents, immobilisation, hydrogels, supports

## Abstract

This review focuses on the synthesis of plant-mediated zinc oxide nanoparticles (ZnO NPs) and their applications for antibacterial and photocatalytic degradation of dyes, thereby addressing the need for sustainable and eco-friendly methods for the preparation of NPs. Driven by the significant rise in antibiotic resistance and environmental pollution from dye pollution, there is a need for more effective antibacterial agents and photocatalysts. Therefore, this review explores the synthesis of plant-mediated ZnO NPs, and the influence of reaction parameters such as pH, annealing temperature, plant extract concentration, etc. Additionally, it also looks at the application of plant-mediated ZnO NPs for antibacterial and photodegradation of dyes, focusing on the influence of the properties of the plant-mediated ZnO NPs such as size, shape, and bandgap on the antibacterial and photocatalytic activity. The findings suggest that properties such as shape and size are influenced by reaction parameters and these properties also influence the antibacterial and photocatalytic activity of plant-mediated ZnO NPs. This review concludes that plant-mediated ZnO NPs have the potential to advance green and sustainable materials in antibacterial and photocatalysis applications.

## 1. Introduction

Over the years, there has been a global upsurge in the interest in environmentally friendly and sustainable nano-synthesis approaches. These synthesis approaches fall under green synthesis and their key focus is on reducing and eliminating the need for hazardous and harmful chemicals in nanoparticle (NP) production. One such approach that has gained significant interest in the scientific community is green synthesis using plant materials. Its attractiveness stems from its simplicity, environmental friendliness, and cost-effectiveness. Moreover, plant-mediated nano-synthesis produces biocompatible NPs that can be employed for a wide range of applications [1]. Therefore, it is a viable alternative to chemical and physical nano-synthesis methods such as pulsed laser deposition, infrared irradiation, sputtering, chemical vapour deposition, etc. Even though some of these chemical and physical nano-synthesis methods are widely used, they face drawbacks which include the use of hazardous chemicals, the need for pressure and temperature control, high energy consumption, high cost, and time constraints, which impact the environment negatively [2]. Table 1 shows some of the advantages and disadvantages of green synthesis of ZnO NPs [3].

Plant-mediated ZnO NPs have been extensively studied owing to their remarkable performance in various applications. Plants such as *Pluchea indica* [4], *Cnidoscolus aconitifolius* [5], *Mucuna pruriens* [6], *Scoparia Dulcis* [7], *Tilia Tomentosa* [8], *Azadirachta indica* (*A. indica*) [9,10], *Zingiber Officinale* (*Z. officinale*) [11] etc., have been successfully used to prepare ZnO NPs for various applications including antibacterial, anticancer, antioxidant, supercapacitors, photodegradation of dyes, agriculture, gas sensing, glucose sensing, solar cells, and sunscreen. Among the many applications of plant-mediated ZnO NPs applications, photocatalytic degradation of dyes and antibacterial applications are the most explored and this is owing to their outstanding performance that has been well documented in the literature [12,13]. Dyes are used in many industries such as fabric, pharmaceutical, food, tanneries, leather, cosmetics, etc., and large amounts of these dyes make up part of industrial wastewater. Conventional wastewater treatment technologies are used to treat industrial wastewater; however, one of the major drawbacks of some of these technologies is their failure to treat dye wastewater adequately [14]. This is a major concern because discharging untreated or inadequately treated dye effluents into waterbodies poses a serious threat to human lives, aquatic lives, and the environment. Photodegradation of dyes using semiconductor metal oxides such as ZnO NPs offers an alternative remedy that is consistent, secure, and capable of degrading dyes even at low concentrations [12,15]. Additionally, there has been a global surge in antibiotic drug resistance, posing a threat to the health of animals and humans, as well as food production [16]. Therefore, new technologies must be developed to combat this threat. Plant-mediated ZnO NPs have demonstrated outstanding antibacterial activity against a wide range of bacteria and, therefore, are a potential remedy to combat antibiotic drug resistance [17].

In light of these, this review delves into the synthesis of ZnO NPs using plant materials and their applications in the photocatalytic degradation of organic dyes and antibacterial activity against a wide spectrum of bacteria. The review will highlight the influence of synthesis parameters such as pH, annealing temperature, reaction temperature, plant extract, and Zn salt precursor concentrations on the properties of plant-mediated ZnO NPs such as crystallite size, morphology, and optical bandgap. The influence of properties of plant-mediated ZnO NPs and reaction parameters such as initial pH, catalyst dosage, and initial dye concentration on the photodegradation efficiency will be discussed in detail. Additionally, the antibacterial mode of action of plant-mediated ZnO NPs will be analysed. Lastly, conclusions and recommendations will be given.

## 2. ZnO NPs Properties and Their Importance

In recent years, ZnO NPs have sparked major research and industrial interest owing to their numerous properties which allow their applications in various industries. For instance, ZnO NPs have been explored for gas sensing owing to their high chemical sensitivity, high electron mobility of 200 cm^2^/V.s, and high thermal stability [18]. Their high exciton-binding energy of 60 meV has led to their use in photonic devices such as light-emitting devices (LEDs), photodiodes, and phototransistors [19]. The properties of ZnO NPs like transparency, low adhesiveness, and ability to absorb ultraviolet (UV) light have allowed their use in cosmetics [20]. ZnO NPs absorb electromagnetic radiation and exhibit photoluminescence, resulting in their application in field emission display devices. In the rubber industry, ZnO NPs are used as additives to enhance the properties of rubber such as strength, antiaging, and toughness, while their wide bandgap of 3.37 eV at room temperature (RT) results in their applications in optoelectronic devices such as supercapacitors [21,22]. Table 2 shows some of the physiochemical properties of ZnO NPs [23,24].

Additionally, ZnO NPs possess antibacterial, anticancer, antioxidant, and anti-inflammatory properties in addition to being non-toxic and biocompatible, thus making ZnO NPs one of the sought-after metal oxides in the biomedical field [25]. Moreover, their ability to absorb UV light and generate electron–hole pairs has led to their applications as photocatalysts for the photodegradation of organic dyes and hydrogen gas production through water-splitting [26,27].

## 3. Synthesis of ZnO NPs

**Figure 1:**
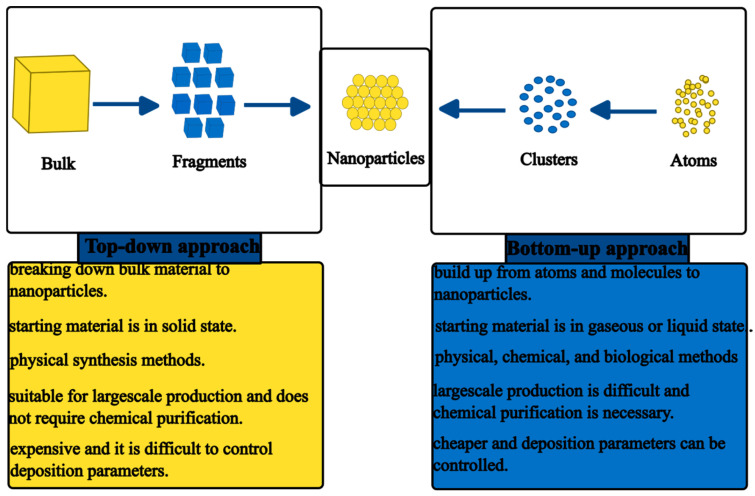
Contrast between the top-down and bottom-up synthesis approaches.

Several synthesis methods for the preparation of ZnO NPs have been reported in the literature [28,29]. Generally, these methods can be divided into top-down and bottom-up approaches. The top-down approach involves the physical or chemical breakdown of bulk materials to NPs, while the bottom-up approach utilises smaller particles such as atoms, ions, and molecules to build up the NPs [30]. Figure 1 shows the contrast between the top-down and bottom-up approaches of synthesising NPs [31].

**Figure 2:**
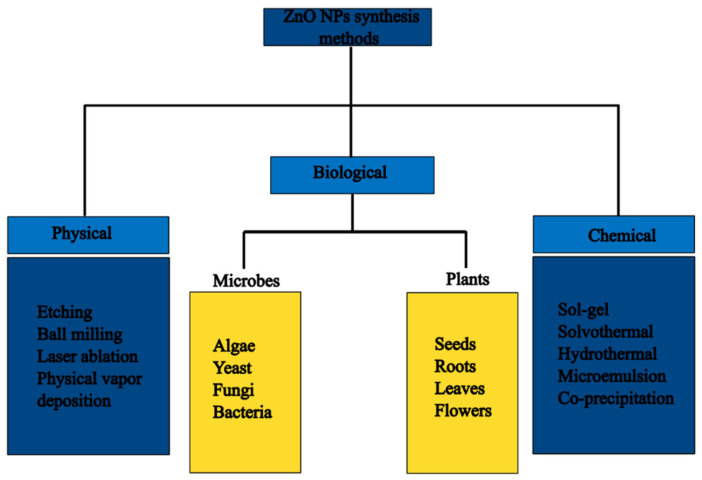
An overview of ZnO nanoparticle synthesis methods.

The approaches can be further subdivided into three categories, which are physical, chemical, and biological/green synthesis methods, as shown in Figure 2.

### 3.1. Physical Synthesis Methods of ZnO NPs

Physical methods such as laser ablation, ball milling, thermal evaporation, sputtering, arc discharge, and molecular beam epitaxy are some of the synthesis methods that have been employed to fabricate ZnO NPs [32,33]. These methods are ideal for the preparation of ZnO NPs on an industrial scale owing to their high production rates. However, the physical synthesis methods for NPs are hampered by the need for vacuum, exposure to radiation, and high operating temperatures which require cooling systems [34]. This consequently leads to high costs due to the need for specialised equipment and the need for skilled personnel for the operation of the equipment. Some of the physical methods that have been utilised for the preparation of ZnO NPs are discussed below.

Laser ablation is a physical synthesis method that involves targeting a laser on the surface of precursor material in a liquid or gaseous environment. When the precursor material is heated by the laser, ablation occurs leading to vapour or plasma formation, depending on the intensity of the laser beam. The NPs are formed from the rapid cooling and condensing of the vapour or plasma. The process can occur using a continuous laser (laser ablation) or pulsed laser (pulsed laser ablation) [35]. The advantages of the laser ablation method include not requiring harsh and toxic solvents, being safe, and producing NPs with narrow particle sizes. However, it faces several challenges such as high cost, low yield, and difficulties in controlling the morphology and structure of the NPs [36,37]. Ismail et al. [38] prepared high-purity colloidal ZnO NPs using Nd:YAG laser ablation at RT by targeting Zn metal in water. The laser energy density (laser fluence) influenced the properties of the synthesised ZnO NPs such as optical and particle size, with particle size increasing with an increase in laser energy density.

High ball milling is a physical synthesis method that is commonly used for the synthesis of ZnO NPs. The method is based on targeting bulk material with high-energy balls, which transfers the kinetic energy from the balls to the bulk material, leading to the breaking down of bonds in the bulk material. This results in smaller particles breaking away with newer surfaces from the bulk material [39]. While ball milling is a convenient and efficient method used to produce NPs even on an industrial scale, it is hampered by high energy demands and lengthy synthesis times. Salah et al. [40] prepared spherical ZnO NPs using the high-energy ball milling method. The results from transmission electron microscopy (TEM) revealed that ZnO NPs produced after 50 h of milling had particle sizes of about 30 nm. These results were in agreement with the scanning electron microscopy (SEM) results. The particle size of the synthesised ZnO NPs decreased with an increase in milling time. Additionally, the obtained ZnO NPs were reported to be ideal for antibacterial applications due to their structural and optical properties.

Sputtering is an efficient physical method used to produce thin-film nanostructures by targeting a solid surface with high-energy gas or plasma particles [41]. The bombardment of the solid surface using high-energy gaseous ions causes the physical ejection of clusters of atoms [42,43]. Generally, the sputtering process can be summarised in four steps. The first step involves the formation of the positively charged gaseous ions followed by the bombardment of the cathode target by the positively charged gaseous ions due to electric field acceleration. The third step involves the ejection of particles from the cathode target and finally formation of a thin film on the substrate due to condensation. The sputtering process can be achieved using three different power sources or a combination of the three, which are magnetron, radio-frequency (RF) diodes, and direct current (DC) diodes [44,45].

Gridchin and associates [46] utilised the RF magnetron sputtering method for the preparation of ZnO thin films. The deposition was conducted in Ar/O_2_ plasma gas with varying O_2_ compositions and growth temperatures. The authors observed that the ZnO thin films synthesised in the absence of O_2_ with pure Ar plasma gas were porous, while the thin films synthesised at 200–300 °C were textured and polycrystalline. The sputtering deposition method can be utilised for a wide selection of materials, e.g., metals, metal oxides, insulators, semiconductors, and organic compounds. However, some of its drawbacks include the need for a larger target, the high cost of the target and equipment, and low purity [47]. Table 3 gives some advantages and disadvantages of some physical synthesis methods used to prepare ZnO NPs. 

### 3.2. Chemical Synthesis Methods of ZnO NPs

Chemical methods such as sol–gel, solvothermal, hydrothermal, microemulsion, and chemical vapour deposition are some of the synthesis methods that have been utilised for the preparation of ZnO NPs. Wet chemical synthesis methods are the most studied and reported synthesis methods for the preparation of NPs. However, the chemical synthesis methods are associated with problems with agglomeration of NPs during synthesis. To address this problem, capping agents or stabilisers are added to prevent agglomeration as well as to control the particle size [32]. Capping agents such as polyvinylpyrrolidone (PVP), cetyltrimethylammonium bromide (CTAB), polyethylene glycol (PEG), and sodium dodecylbenzene sulfonate (SDS) are used in several wet chemical synthesis methods. The major drawback associated with the use of some of the capping agents is their non-biodegradability and toxicity [50]. The use of chemical methods enables the preparation of ZnO NPs with diverse properties and applications, and some of the methods are discussed below.

Sol–gel is an attractive and versatile method that is used for the preparation of metal oxide NPs. Decades ago, the sol–gel method was used in ceramic and glass material production; it was later adopted for NP synthesis. It involves the formation of solid particles in a liquid called ‘sols’ followed by the formation of a network of particles enclosed in a liquid called ‘gels’ through crosslinking of the sols. Sol–gel occurs through two main processes, which are hydrolysis and condensation, as shown in Equations (1) and (2) [37,51].

(1) Hydrolysis: M−OR+HOH→M−OH+R−OH

(2) Condensation: M−OH+XO−M→M−O−M+XOH

where M is a metal, R is an alkyl group, and X is H or an alkyl group.

The hydrolysis process occurs in water or alcohol using precursors such as metal alkoxides and metal salts, which leads to the formation of a sol. This is followed by condensation which involves the formation of metal–hydroxy–metal bond (M−OH) and metal–oxo–metal bond (M−O−M) bridges through olation and oxolation processes, respectively [52]. The condensation process is followed by ageing, drying to remove water and alcohol from the gel, and lastly, calcination to obtain the NPs. Reaction parameters, such as pH, hydrolysis time, type of precursor used, ageing time, and water-to-precursor ratio, have an impact on the structure, particle size, morphology, and other properties of the synthesised NPs [53]. Therefore, controlling these parameters during synthesis can help tailor the properties of the NPs for specific applications.

For instance, ZnO NPs were prepared by the sol–gel method under acidic conditions using Zn(NO_3_)_2_·6H_2_O as the precursor, isopropyl alcohol as a solvent, and glycerine as a stabiliser. The reaction was carried out at 70 °C for 2 h and dried at 70 °C for 24 h. The obtained gel was calcined at varying temperatures, at 500, 700, and 900 °C. The synthesised ZnO NPs were observed to be of irregular morphology and with particle sizes ranging from 50 to 100 nm based on the field emission scanning microscopy (FESEM) results. The authors also observed that particle size increased as the calcination temperature increased [54]. In another study, ZnO NPs were prepared by the sol–gel method under acidic conditions using zinc acetate dihydrate, sodium hydroxide, and PEG as precursors and calcined at 800 °C. The obtained ZnO NPs were observed to be spherical with a particle size distribution between 50 and 70 nm from the TEM images [55].

Another chemical method that has been widely utilised for the synthesis of NPs is the hydrothermal synthesis method. It involves the interaction of an aqueous solvent with the precursor usually metal salts at medium to high temperature and pressure in an autoclave. When the process takes place in a non-aqueous environment, with a non-aqueous solvent, it is called solvothermal [56,57]. The synthesis parameters of the hydrothermal/solvothermal method influence the structure, particle size, and morphology which results in different properties of the synthesised NPs [58,59]. However, difficulties in controlling the particle size of NPs during synthesis and issues with reproducibility are some of the limitations of hydrothermal methods [60]. Wang et al. [61] utilised the solvothermal method for the preparation of ZnO NPs for the photodegradation of methyl orange (MO) and p-nitrophenol. The ZnO NPs were synthesised using zinc acetate dihydrate, sodium hydroxide, and ethanol. The reaction mixture was heated at 80 °C for 2 h and transferred to an autoclave for 12 h at 180 °C. The prepared ZnO NPs were observed to be near-spherical with a particle size diameter of about 25–40 nm from the SEM images.

The chemical reduction synthesis method has been widely studied in research laboratories for the preparation of a wide range of NPs. This is due to its ability to produce NPs of various properties and morphologies. The technique involves the reduction of metal salt precursors such as nitrates, sulphates, and chlorides using reducing agents such as sodium triethyl borohydride, sodium borate, toluene, sodium hydroxide, and hydrazine. The reduction process leads to the formation of metallic atoms or metal oxide in the form of a precipitate. The NPs can be stabilised using capping agents to prevent aggregation [62]. For instance, ZnO NPs were prepared by the chemical reduction of zinc nitrate hexahydrate using sodium hydroxide and starch as the stabilisation agent. The synthesised ZnO NPs were analysed using TEM and SEM. The ZnO NPs were revealed to have an average particle size of 48 nm and spherical with a few hexagonal particles, respectively [63]. The chemical reduction method has several drawbacks which limit its application, and these include toxicity of some of the reducing agents, relative costliness, issues with impurities, and poor reduction [64]. Table 4 depicts some of the advantages and disadvantages of some chemical synthesis methods of ZnO NPs.

### 3.3. Green Synthesis

Green chemistry has been defined by the United States Environmental Protection Agency (EPA) as “an area of chemistry, chemical engineering or/and related fields focused on the designing of chemical/products and/or processes, which reduce/decreases or remove the utilisation and/or creation of dangerous/toxic substances” [69]. The green synthesis of NPs using plants, algae, and microbes is a green chemistry technology as it eliminates the need for toxic raw materials and minimises the production of toxic products. Therefore, it lessens the negative impacts of chemical synthesis technologies on the environment and human health. Additionally, green synthesis of NPs reduces energy consumption and is a cost-effective method [70]. It is an alternative method to the conventional synthesis methods that has been receiving attention in recent years. Green synthesis can be divided into microbes, plants, and algae, with bacteria, yeast, and fungi falling under microbes.

Biomolecules such as enzymes, proteins, and carbohydrates from or in microbes are capable of reacting with metallic ions via reduction or oxidation to produce NPs [71]. These biomolecules remove the need for toxic reducing agents and capping agents as they act as reducing and stabilising agents. The microbes interact with the metallic ions via several pathways which can affect the size and morphology of the NPs. Moreover, the size and morphology can be manipulated by controlling synthesis conditions such as pH and temperature [72]. The reactions can be intracellular or extracellular, with intracellular requiring extra costly purification processes to obtain the synthesised NPs from the cells. Bacteria are microorganisms that are abundantly found in the environment, water, soil, and land. They are capable of thriving even under severe conditions and reproducing, making them suitable candidates for NP production [73]. Bacteria are known to produce biomolecules such as enzymes, carbohydrates, proteins, and amino acids through various metabolic pathways. These biomolecules play an essential role in the growth, reproduction, and adaptability of the bacteria to the environment. Recently, the biomolecules from bacteria have found use in the fabrication of NPs. The biomolecules are capable of reducing metal ions to generate NPs and act as stabilising agents that can prevent aggregation during the synthesis process [74].

Barani et al. [75] utilised two bacteria strains isolated from water and shrimp (*Marinobacter* sp. 2C8 and *Vibrio* sp. VLA) for the synthesis of ZnO NPs using zinc sulphate monohydrate as the Zn salt precursor. The obtained bacteria strains from the isolation process were incubated at 30 °C for 72 h in broth media to obtain bacterial biomass. The biomass was incubated at 30 °C for 48 h and centrifuged to collect the supernatant that was utilised for the synthesis of ZnO NPs. The TEM results revealed that the fabricated ZnO NPs were roughly spherical with average particle size diameters of 10.23 and 20.26 nm for *Marinobacter* sp. 2C8-mediated ZnO NPs and *Vibrio* sp. VLA-mediated ZnO NPs, respectively.

Amongst microorganisms used for the green synthesis of NPs, fungi-mediated synthesis has been reported to exhibit better performance. This is owing to the presence of a variety of intracellular biomolecules and biomolecules on the surface of the cells. Moreover, fungi are capable of producing larger amounts of NPs compared to bacteria, and this makes fungi-mediated synthesis more ideal for the large-scale production of NPs [76,77]. Kalpana and co-workers [78] successfully synthesised ZnO NPs using *Aspergillus niger* fungi and zinc nitrate at 32 °C for 48 h with shaking; the authors observed a colour change from light yellow to colourless. The morphology of the synthesised ZnO NPs was studied using SEM and reported to be spherical with an average particle size of 61 nm. Additionally, the authors confirmed the presence of ZnO NPs and the functional groups such as carboxylic and aromatic groups on the surface of the synthesised ZnO NPs using Fourier transform infrared spectroscopy (FTIR). The functional groups were attributed to be from the fungi biomolecules.

NPs of different morphologies and sizes have been successfully synthesised using microorganisms. This synthesis technique is a green synthesis route that avoids the use of toxic chemicals and synthesises NPs in an eco-friendly manner. However, the method is limited by the need for screening, long incubation periods, the need for high aseptic conditions, and complex maintenance [79]. Additionally, there is a risk of contamination of the synthesised NPs with the microorganisms posing a threat to human health. Green synthesis of NPs using plants offers a more favourable option and has been explored for producing ZnO NPs with excellent results.

### 3.4. Plant-Mediated Synthesis

Plants are abundant and easily accessible natural resources that are “chemical factories” and have a vast majority of applications. For instance, they are employed in environmental remediation to eliminate heavy metals and other pollutants from the environment through processes like detoxification, accumulation, or phytoremediation. Plants require little maintenance as compared to microbe-mediated green synthesis, and similar to microbes, plants possess biomolecules which can be used to fabricate NPs. Moreover, the reaction kinetics during plant-mediated synthesis is comparable to chemical synthesis methods [80]. This makes plant-mediated synthesis of NPs a viable option for the environmentally friendly and sustainable nano-synthesis of NPs.

Plant parts such as leaves, roots, stems, flowers, and fruits produce large quantities of biomolecules via various metabolic pathways that can be used to fabricate NPs. The biomolecules have free radical-scavenging capabilities and are rich in antioxidant activity [81]. The biomolecules include vitamins, proteins, terpenoids, reducing sugars, polyphenols, polyols, etc., which can be utilised to reduce metal salts to NPs and stabilise the NPs. Green synthesis of NPs using plant materials has been extensively studied and reported owing to its ability to be easily scaled up for large-scale production, cost-effectiveness, and environmentally friendly nature [82]. Even though plant-mediated synthesis of NPs has several advantages over chemicals and microbe-mediated synthesis, research is still required to better control the size and morphology of the NPs.

#### Plant-Mediated Synthesis of ZnO NPs

ZnO NPs have been synthesised using different plants and plant parts, with different zinc salts such as nitrates, chlorides, sulphates, and acetates as the Zn precursors. The synthesis process can be divided into three steps: plant collection and treatment, biomolecule extraction, and synthesis. The process begins with the collection of fresh plant material which is followed by washing of the plant material to remove dirt and debris. The washed plant material is dried or used in its fresh form. The drying process can be achieved using different drying techniques like sun-drying, oven-drying, and freeze-drying. Typically, drying is preferred as it eliminates moisture and stops any metabolic processes, which in turn aids in the reproducibility of the synthesis process. After drying, the plant material is ground to powder to increase the interaction with the solvent during the extract process.

The next step is the biomolecule extraction step which involves the extraction of the biomolecules from the dried powder or small pieces of fresh plant material, typically using non-toxic solvents such as water and ethanol. The extraction is usually carried out at low temperatures (RT to 80 °C) using conventional extraction methods such as maceration, decoction, and Soxhlet extraction. In some cases, non-conventional extraction methods such as microwave-assisted, ultrasound-assisted, autoclave-assisted extraction, etc., are utilised for the extraction of biomolecules from plant material. Chennimalai et al. [83] utilised a combination of maceration and heat-assisted extraction to extract biomolecules from *Opuntia humifusa* (*O. humifusa*) fruit. The extraction process entailed adding 100 mL of distilled water to 20 g *O. humifusa* fruit powder, shaking the mixture for 24 h and heating the mixture in an oven at 60 °C. The qualitative analysis of the extract yielded saponins, polyphenols, tannins, flavonoids, terpenoids, carbohydrates, and proteins. These biomolecules have been reported to take part in the plant-mediated synthesis of ZnO NPs.

**Figure 3:**
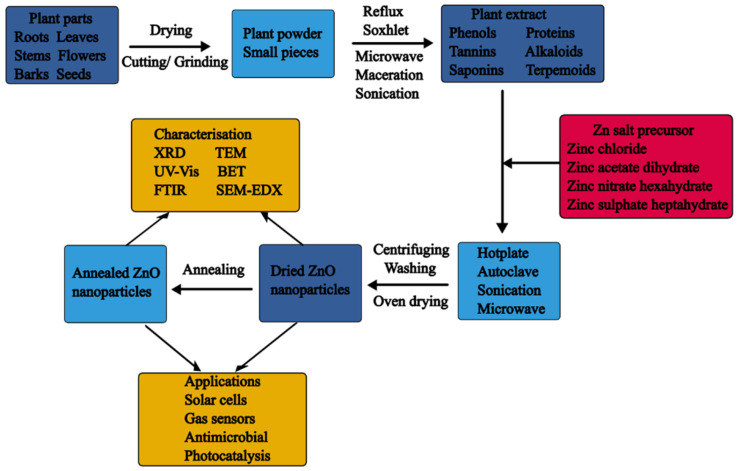
Summary of plant-mediated synthesis of ZnO NPs.

After obtaining the plant extract, it can be mixed with the Zn salt precursor and heated at low temperatures (usually from RT to 100 °C), which is one of the appeals of plant-mediated synthesis. The reduction of the Zn salt precursors and the stabilisation of the synthesised ZnO NPs by the plant biomolecules occurs in this step and the mechanism will be discussed in detail in later sections. Typically, the plant-mediated synthesis of ZnO NPs has followed two common approaches: co-precipitation and direct reduction. In co-precipitation, two precursors are used, hydroxides like sodium hydroxide and potassium hydroxide and the Zn salt precursors. In comparison, the direct reduction approach involves the reduction of the Zn salt precursors using the plant extract. A summary of plant-mediated synthesis of ZnO NPs is depicted in Figure 3.

Sathappan and associates [84] successfully synthesised ZnO NPs with *Cissus quadrangularis* (*C. quadrangularis*) stem extract and zinc acetate dihydrate. The method involved mixing 1 mL of *C. quadrangularis* extract with 50 mL of 0.02 M zinc acetate dihydrate and adjusting the pH to 12 using 2 M sodium hydroxide. The mixture was allowed to stand for 2 h, and a white precipitate was obtained, centrifuged, and washed with ethanol. To obtain the ZnO NPs, the white precipitate was dried in the oven at 60 °C overnight. In another study, *Hertia intermedia* (*H. intermedia*) extract was utilised to fabricate ZnO NPs. The procedure involved adding 10 mL of *H. intermedia* extract to 100 mL of 0.1 M zinc sulphate and maintaining the pH at 8. The mixture was heated at 60 °C for 6 h to obtain a yellowish-brown mixture. The mixture was centrifuged and washed with ethanol and dried at 50 °C in the oven [85]. Donga and Chanda [86] successfully prepared ZnO NPs using 50 mL of *Caesalpinia crista* (*C. crista*) seed extract and 5 g of zinc nitrate hexahydrate at 80 °C. The mixture was heated until the formation of a deep yellow-coloured paste. The paste was annealed at 400 °C for 2 h in a Muffle furnace to obtain the ZnO NPs.

In some instances, the synthesis can be microwave-assisted, ultrasound-assisted, or autoclave-assisted. Algarni and associates [87] successfully synthesised ZnO NPs for photodegradation application using 5 g zinc nitrate hexahydrate, 18 mL sodium hydroxide, and 30 mL *Rosmarinus officinalis* leaf extract in a steel autoclave at 80 °C and 180 °C. Sonication was utilised for the preparation of *Thymus vulgaris* (*T. vulgaris*)-mediated ZnO NPs. The method involved adding *T. vulgaris* to 0.1 M zinc nitrate hexahydrate in a 4:1 ratio and sonication for 1 h to obtain a white-coloured precipitate. The precipitate was washed with ethanol and deionised water, dried in the oven at 50 °C, and annealed in the furnace at 400 °C [69]. In another study, a microwave-assisted method was used for the synthesis of ZnO NPs using *Rumex dentatus* (*R. dentatus*) leaf extract. The method involved adding 100 mL of 0.1 M zinc nitrate hexahydrate to 100 mL of *R. dentatus* extract on a hotplate at 60–70 °C with stirring. This was followed by microwave treatment of the reaction mixture at 500 W for 5 min in a domestic microwave. The mixture was separated and washed, and the obtained paste was dried in the oven at 50 °C for 4 h [88].

A few studies have used solution combustion to prepare plant-mediated ZnO NPs. This approach involves heating a mixture of the plant extract and the Zn salt precursor in a furnace. Surendra et al. [89] prepared *Lantana camara* (*L. camara*) leaf-mediated ZnO NPs by adding 8.99 g of zinc nitrate hexahydrate to 5 mL of *L. camara* extract and heating the mixture at 80 °C for 10 min on a heating mantle. The mixture was then subjected to 500 °C (combustion) to obtain the ZnO NPs in a furnace.

### 3.5. Influence of Synthesis Parameters on the Formation and the Properties of Plant-Mediated ZnO NPs

This section will discuss the influence of synthesis parameters on the formation and properties of plant-mediated ZnO NPs. The synthesis parameters such as pH, reaction time, precursor salt concentration, temperature, type of Zn salt precursor, and the influence of the type of biomolecules present in the extract and plant extract concentrations play a role in the formation of plant-mediated ZnO NPs and hence their properties [90].

The concentration of salt precursor is known to influence the properties of NPs during synthesis. Generally, increasing the Zn precursor concentration to a certain point (optimum) results in increased particle rate growth, which leads to the formation of smaller particles. However, any further increase in Zn precursor concentration beyond the optimum precursor concentration leads to agglomeration, resulting in larger particles [91]. This trend was observed by Abdol Aziz and co-workers [92] for ZnO NPs prepared with *Musa acuminata* (banana) peel extract and varying concentrations of zinc acetate dihydrate (0.01, 0.05, and 0.1 M). The authors observed that the crystallite size decreased with an increase in precursor concentration from 0.01 to 0.05 M (25.78 to 14.93 nm, respectively) from the X-ray powder diffraction (XRD) results. Nevertheless, a further increase in precursor concentration to 0.1 M increased the crystallite size to 15.23 nm. Additionally, increasing the Zn salt precursor concentration resulted in a decrease in the calculated bandgap from 3.54 to 3.44 eV. Thus, the calculated bandgap red-shifted towards lower energy values with a decrease in crystallite sizes. However, increasing the Zn salt precursor concentration beyond 0.05 M resulted in an increase in the bandgap to 3.58 eV. As a result of quantum confinement effects, the bandgap is expected to blue shift towards higher energy values with a decrease in crystallite size. However, in the case of banana peel-mediated ZnO NPs, the crystallite sizes exceed the exciton Bohr diameter of ZnO; therefore, the quantum confinement effects are less prominent [93].

In another study, the influence of different salt precursor concentrations (5, 10, and 50 mmol kg^−1^) on the size and morphology of aloe vera-mediated ZnO NPs was investigated. The XRD results revealed that increasing the Zn salt precursor resulted in a slight increase in crystallite size, while the TEM results showed that varying the precursor salt concentration affected the shape and particle size of the aloe vera-mediated ZnO NPs. The aloe vera-mediated ZnO NPs synthesised with 5, 10, and 50 mmol kg^−1^ exhibited hexagonal, spherical and hexagonal, and cuboidal/rod-shaped particles, respectively. The average particle sizes were 63 nm, 65 nm and 60–180 nm, and 40–45 nm, respectively [94]. These findings demonstrate that Zn precursor salt concentration influences the formation and properties of plant-mediated ZnO NPs. They also highlight that the Zn salt precursor concentration can be used to manipulate and control properties such as the shape and size of plant-mediated ZnO NPs, which can lead to diverse applications of the plant-mediated ZnO NPs.

The use of different Zn salt precursors has been reported to influence the properties of plant-mediated ZnO NPs. This is supported by the investigations by Fakhari and associates [95] who utilised *Laurus nobilis* (*L. nobilis*) leaf extract and zinc acetate and zinc nitrate precursors to prepare ZnO NPs. From the SEM images, the authors observed that the ZnO NPs from the zinc acetate precursor were spherical like bullets, whereas the ZnO NPs from the zinc nitrate precursor were nano-flowers. The spherical ZnO NPs had a smaller particle size (21.26 nm) compared to the nano-flowers (25.49 nm). In a similar study, Abdullah and co-workers [96] assessed the influence of different Zn salt precursors on the properties of *Phoenix dactylifera* (*P. dactylifera*)-mediated ZnO NPs. The authors observed that the choice of Zn salt precursor resulted in different particle sizes and shapes of the synthesised ZnO NPs from the SEM and TEM results, with the different morphologies observed given in Table 5.

These investigations show that the type of Zn salt precursor used can influence the size and shape of plant-mediated ZnO NPs. Therefore, Zn salt precursors can be a tool to control and tailor the morphology of plant-mediated NPs for specific applications.

**Figure 4:**
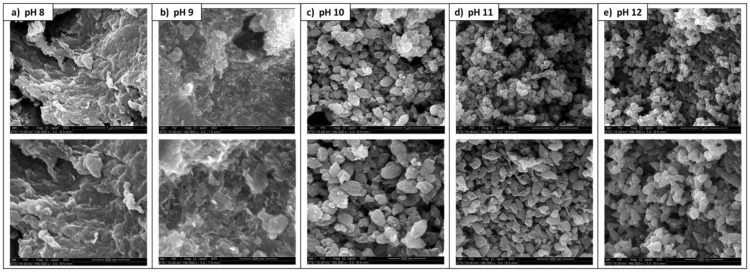
SEM images of *M. acuminata* peel extract-mediated ZnO NPs synthesised at different pH. Adapted from Ref. [97].

pH is an important parameter in nano-synthesis, and this also applies to plant-mediated synthesis of ZnO NPs. However, the influence of pH on the synthesis and properties of plant-mediated ZnO NPs has not been extensively investigated at the present moment. Among the few studies that have investigated the influence of pH on the synthesis and properties of plant-mediated ZnO NPs, several of the studies have proposed pH 12 as the ideal pH for the synthesis of ZnO NPs. Abdullah et al. [97] investigated the influence of pH on the crystallite size and morphology of *M. acuminata* peel-mediated ZnO NPs. The ZnO NPs were synthesised at varying alkaline pHs of 8, 9, 10, 11, and 12. The SEM results (Figure 4) revealed that the ZnO NPs synthesised at pH 8, 9, 10, 11, and 12 were granulated and crumbled, flaked and sheet-like, leaf-like, triangular-like, and triangular-like with tapered tips, respectively. From the XRD results, the authors observed that the *M. acuminata*–ZnO NPs synthesised at pH 8, 9, and 10 were wurtzite in structure with some zinc hydroxide impurities, whereas those synthesised at pH 11 and 12 were high-purity ZnO NPs with a wurtzite structure.

The crystallite size decreased significantly from 79.9 to 30.2 nm with an increase in pH from 8 to 12. The authors proposed pH 12 as the optimum pH for the synthesis of *M. acuminata*-mediated ZnO NPs. Similarly, Gupta [98] and associates optimised the pH for the synthesis of ZnO NPs using *Catharanthus roseus* (*C. roseus*) extract using UV-Vis. The authors reported that *C. roseus*-mediated ZnO NPs synthesised at pH 12 exhibited an absorption peak at 366 nm, whereas no peaks were observed for those prepared at pH 8, 10, and 14. These findings suggest that the ideal pH for synthesising *C. roseus*-mediated ZnO NPs was pH 12. This can be attributed to the presence of the optimum ratio of hydroxyl ions to hydrogen ions at pH 12 which promotes the formation of the Zn–O bond as a result of the attraction between the positively charged zinc (II) ions and the hydroxyl ions. Therefore, at low pH, there are insufficient hydroxyl ions to facilitate the formation of the Zn–O bond, whereas at higher pH, there are excess hydroxyl ions resulting in the Zn–O bond formation [99]. Hence, pH 12 has been utilised in several reports for the synthesis of ZnO NPs using plant extracts.

However, some studies have reported pH 8 as the ideal pH for the synthesis of ZnO NPs using plant materials. Gherbi et al. [100] synthesised ZnO NPs using *Portulaca oleracea* (*P. oleracea*) leaf extract at different pHs (4, 6, 9.5, and 11) by adding sodium borohydride. The authors reported that the XRD calculations showed an increase in the crystallite size of ZnO NPs (25.38 to 26.04 nm) when the pH increased from pH 4 to 6. In contrast, a decrease in crystallite size (25 to 22.17 nm) was observed in the ZnO NPs prepared under alkaline pH conditions, from pH 9.5 and 11. Moreover, the XRD results revealed the presence of impurities in the ZnO NPs synthesised at pH 4 and 11. Therefore, the authors proposed that pH 8 was the ideal pH for the preparation of *P. oleracea*-mediated ZnO NPs of high purity and smaller sizes.

In another study, Mohammadi and Ghasemi [101] synthesised ZnO NPs at pH 4, 6, 7, 8, and 10 using cherry extract. The results from the UV-Vis analysis revealed that no absorption peak was observed for cherry-mediated ZnO NPs at pH 4. The absorption peaks red-shifted towards higher wavelengths as the pH increased from 4 to 8 due to larger particles, while a further increase in pH from 8 to 10 exhibited a blue shift towards lower wavelengths due to smaller particles. The authors concluded that the optimum pH for the synthesis of ZnO NPs using cherry extract was 8. It has been suggested that alkaline conditions influence deprotonation and activation of biomolecules which increases their reducing and stabilising power. On the other hand, acidic conditions have been reported to influence protonation, which reduces the reducing and stabilising power of biomolecules present in plant extracts [102].

However, pH has been reported to affect the electrical charges of the biomolecules present in the plant extracts, which in turn affects their power to reduce and stabilise the NPs and hence the growth of the NPs [103]. Therefore, the ideal pH for preparing plant-mediated ZnO NPs should depend on the type of biomolecules present in the plant extracts. Padalia and co-workers [104] investigated the influence of pH 5 and 8 on the particle size of *Salvadora oleoides* (*S. oleoides*)-mediated ZnO NPs. The study revealed that the *S. oleoides*-mediated ZnO NPs synthesised at pH 5 had smaller particle sizes (average 26.62 nm) with spherical particles compared to those synthesised at pH 8 (average 38.62 nm), with irregular morphology from the TEM images. In another study, Sarillana and co-workers [105] synthesised ZnO NPs at pH 7, 8, and 9 using *Theobroma cacao* L. (*T. cacao*) pod husks. The UV-Vis spectra revealed that the absorption peaks of the *T. cacao*-mediated ZnO NPs blue-shifted with a decrease in pH, indicating the formation of smaller particles with a decrease in pH. Therefore, the ideal pH for the synthesis of ZnO NPs using *T. cacao* pod husk extracts was pH 7. These findings demonstrate the influence of pH on the formation of ZnO NPs using plant extracts and therefore, there is a need to optimise the pH as the ideal pH for the synthesis of ZnO NPs using plant extracts might be dependent on the type of biomolecules present in the plant extract. However, only a few studies have investigated the influence of pH on the formation and properties of plant-mediated ZnO NPs and therefore, more studies are required to better understand the influence of pH. This would lead to the development of better plant-mediated ZnO NPs that are suitable for various applications. Additionally, some studies have highlighted the influence of pH on the particle size of plant-mediated ZnO NPs. Therefore, pH can be used as a tool to tune or control the properties of plant-mediated ZnO NPs, leading to plant-mediated ZnO NPs of various properties that are suitable for various applications.

**Figure 5:**
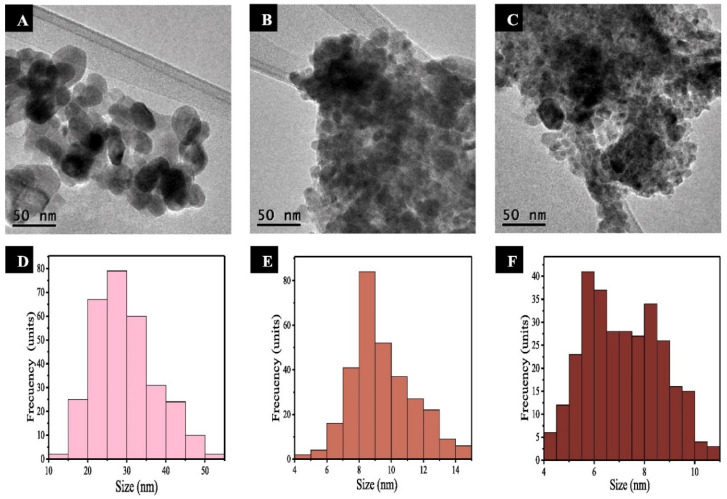
The TEM images of *H. sabdariffa*-mediated ZnO NPs synthesised using (**A**) 1%, (**B**) 4%, and (**C**) 8%. Particle size distribution of H. sabdariffa-mediated ZnO NPs synthesised using (**D**) 1%, (**E**) 4%, and (**F**) 8%. Adapted from Ref. [107].

Plant extract concentration has been reported to influence the properties of plant-mediated ZnO NPs. Nithya and associates [106] investigated the effect of varying the volume of *Cardiospermum halicacabum* (*C. halicacabum*) extract from 0.2 to 0.6 mL on the properties of the synthesised ZnO NPs. The authors reported that increasing the volume of *C. halicacabum* extract resulted in a significant reduction in crystallite size from 62 to 48 nm of the synthesised ZnO NPs from the XRD studies. This was in agreement with the observed dynamic light scattering (DLS) results; the particle size also decreased with an increase in *C. halicacabum* extract volume. This is similar to observations by Soto-Robles and associates [107] for different *Hibiscus sabdariffa* (*H. sabdariffa*) plant extract concentrations used for the synthesis of ZnO NPs. The authors observed from the TEM images that the particle size decreased as the *H. sabdariffa* concentration increased from 1% (20–40 nm) to 8% (5–12 nm). The TEM images and the size distribution are given in Figure 5. The calculated bandgap from the UV-Vis results revealed that the bandgap decreased (2.96 to 2.77 eV) with an increase in *H. sabdariffa* concentration.

Similar to the influence of Zn salt precursor concentration, studies have revealed that increasing the plant extract concentration beyond the optimum leads to an increase in size. This was reported for ZnO NPs synthesised using varying volumes of *Solanum nigrum* (*S. nigrum*) leaf extract. It was observed from the XRD studies that the crystallite decreased from 2.53 to 2.07 nm with an increase in *S. nigrum* volume from 5 to 15 mL. On the contrary, a further increase in *S. nigrum* extract to 20 mL sizes led to an increase in crystallite size to 2.70 nm [108]. At lower plant concentrations, the biomolecules present in the extract are insufficient to reduce and stabilise the synthesised NPs which leads to aggregation and larger particles. On the other hand, excess biomolecules in extracts can affect the nucleation step, also resulting in larger particles. These findings highlight the influence of plant extract volume or concentration on the properties of plant-mediated ZnO NPs. Plant extract concentration can be utilised as a tool for controlling the properties of plant-mediated ZnO NPs as varying this parameter results in varying properties of plant-mediated ZnO NPs.

Temperature is also an important parameter during the synthesis of NPs. The general trend is that increasing reaction temperature decreases the particle size, and this is due to nucleation being favoured over particle growth at high temperatures, which leads to smaller particles. This is because, at high temperatures, the molecules interact with the precursor faster due to their increased kinetic energy, which results in nuclei formation instead of particle growth. However, at low temperatures, the kinetic energy is low, which leads to particle growth due to the Oswald ripening thermal effect. It should be noted that when the temperature is too high, the surface activity of the nuclei increases, which leads to collisions and agglomeration, resulting in increased particle sizes [109]. On the contrary, Basri et al. [110] reported a smaller particle size diameter of pineapple peel-mediated ZnO NPs synthesised at 28 °C (at RT) (8–45 nm) which were a mixture of spherical and nanorods compared to those synthesised at 60 °C (73–123 nm), which were flower rod-shaped. Another study reported an increase in particle sizes of cherry-mediated ZnO NPs with an increase in reaction temperature from RT to 90 °C from the SEM results. The authors attributed these observations to the reduction in stability of biomolecules present in the cherry extract as the temperature increased [101]. This resulted in the cherry extract losing its reducing and stabilising power as temperature increased.

Similar to the reaction temperature, annealing temperature has also been reported to influence the size, morphology, and optical bandgap of ZnO NPs. Doan Thi and associates [111] studied the influence of different annealing temperatures (unannealed and 300 to 900 °C) on the crystallite size of orange peel-mediated ZnO NPs. From the XRD results, the unannealed ZnO NPs exhibited the smallest crystallite size (12 nm), whereas the ZnO NPs annealed at 300 and 900 °C had crystallite sizes of 22 and 95 nm, respectively. The crystallite size increased with an increase in annealing temperature. The authors attributed this to the re-orientation of particles as a result of an increase in thermal energy, which leads to a reduction in the number of defects in grain boundaries. Additionally, the TEM results revealed that the particle size also increased with an increase in annealing temperature (10–20 nm, 35–60 nm, 70–100 nm, and 200–230 nm for as-prepared ZnO NPs, ZnO NPs annealed at 400, 700, and 900 °C, respectively).

Similar observations were reported by Álvarez-Chimal and associates [112] for *Dysphania ambrosioides* (*D. ambrosioides*)-mediated ZnO NPs annealed at 200, 400, 600, and 800 °C. From the SEM analysis, the authors observed that the particle size increased with an increase in annealing temperature, with average particle sizes of 7 and 14 nm for ZnO NPs annealed at 200 and 400 °C, respectively. The *D. ambrosioides*-mediated ZnO NPs were observed to be quasi-spherical from 200 to 400 °C. However, quasi-spherical and hexagonal prisms were observed from 600 to 800 °C with average particle sizes of 22 and 130 nm for quasi-spherical particles and 70 and 90 nm for hexagonal prisms, respectively.

Karthik et al. [113] investigated the influence of different annealing temperatures (100, 300, and 600 °C) on ZnO NPs synthesised using *Acalypha indica* (*A. indica*) leaf extract. From the XRD results, the authors observed that the *A. indica*-mediated ZnO NPs annealed at 100 °C were amorphous and therefore did not exhibit any diffraction peaks. However, the *A. indica*-mediated ZnO NPs annealed at 300 and 400 °C were crystalline, and their crystallite sizes increased with an increase in annealing temperature (39.34 and 43.63 nm, respectively). The TEM results revealed that the *A. indica*-mediated ZnO NPs annealed at 100 and 300 °C were aggregated and of irregular shape, while the *A. indica*-mediated ZnO NPs synthesised at 600 °C were spherical. The particle sizes also increased with an increase in annealing temperature, and crystallinity was also enhanced at 600 °C. The increase in particle size with an increase in annealing temperature can be attributed to agglomeration, which happens at higher temperatures, and the Oswald ripening effect. The higher the annealing temperature, the higher the activation energy for diffusion, which leads to crystalline grain growth, resulting in larger particles [114]. These studies highlight the influence of annealing temperature on the properties of plant-mediated ZnO NPs, and annealing temperature can be used as a parameter that can be manipulated to control the properties of plant-mediated ZnO NPs. This will lead to the development of better-performing plant-mediated ZnO NPs in their various applications.

The use of different plants or plant parts for the synthesis of ZnO NPs also results in varying properties of the synthesised NPs. Kebede Urge et al. [115] synthesised ZnO NPs under the same reaction conditions using *Z. officinale* root and *Allium sativum* (*A. sativum*) bulb extracts. The UV-Vis results revealed that the absorption peak of *Z. officinale*-mediated ZnO NPs was blue-shifted towards shorter wavelengths compared to *A. sativum*-mediated ZnO NPs. Both synthesised ZnO NPs were revealed to be wurtzite in structure and of high and good crystallinity from the XRD analysis. The crystallite sizes of the *Z. officinale*-mediated ZnO NPs and *A. sativum*-mediated ZnO NPs were 19.8 and 21.94 nm, respectively. This report clearly shows that the use of different extracts influences the size and different properties of ZnO NPs. In another study, Naseer and associates [116] synthesised ZnO NPs under the same conditions using *Cassia fistula* (*C. fistula*) and *Melia azadarach* (*M. azadarach*) extracts. The DLS results revealed that the *M. azadarach*-mediated ZnO NPs had smaller particle sizes (3.62 nm) compared to *C. fistula*-mediated ZnO NPs (68.1 nm). The authors attributed the differences in particle size to differences in biomolecules present in the two plant extracts. The biomolecules present in the *M. azadarach* extract had a better-reducing ability compared to the biomolecules present in the *C. fistula* extract. These studies demonstrate that the properties of plant-mediated ZnO NPs are dependent on the type of biomolecules present in the extract, which is also mostly dependent on the type of plant or plant parts. Therefore, utilising different plant extracts to prepare plant-mediated ZnO NPs can help tailor properties such as the size and shape of the synthesised NPs for different applications. Table 6 gives some properties and applications of plant-mediated ZnO NPs that have been reported.

Green synthesis of ZnO NPs has successfully been achieved using various plants and plant parts. Furthermore, synthesis parameters such as precursor concentration, pH, reaction temperature, and so on influence the formation and properties of plant-mediated ZnO NPs. However, the influence of these synthesis parameters on the formation and properties of plant-mediated ZnO NPs has not been extensively reported. There is a need for more research that focuses on manipulating and tailoring plant-mediated ZnO NPs using synthesis parameters such as pH, reaction temperature, annealing temperature, type of Zn salt precursor, plant concentration, and Zn salt precursor concentration. This will lead to the development of high-performing plant-mediated ZnO NPs that can be suitable for various applications. However, understanding the mechanism of formation is also essential in controlling the properties of plant-mediated ZnO NPs. The mechanism of formation of plant-mediated ZnO NPs will be discussed in the section below. Additionally, studies are needed that focus on reproducibility and scalability of the synthesis processes.

### 3.6. Mechanism of Formation of ZnO NPs

**Figure 6:**
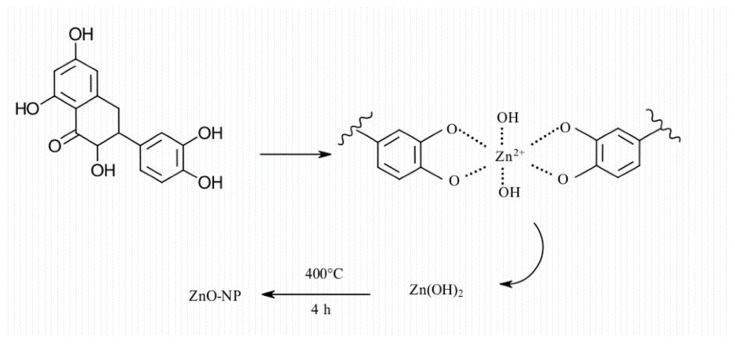
Proposed mechanism of ZnO NP formation using *L. minor*. Adapted from Ref. [117].

Plant extracts have been known to contain biomolecules such as alkaloids, flavonoids, saponins, phenols, terpenoids, tannins, etc. These biomolecules play a significant role in the reduction and stabilisation of ZnO NPs [79]. These biomolecules are also known for their antioxidant properties, which make them ideal for the reduction of Zn salt precursor to ZnO NPs and stabilisation of the ZnO NPs [28]. Del Buono et al. [117] successfully synthesised ZnO NPs using *Lemna minor* (*L. minor*) extract. The phenolic profiles of the *L. minor* extract were screened using ultra-high-pressure liquid chromatography quadrupole time of flight mass spectrometry (UHPLC-ESI/QTOF-MS). The screening yielded tyrosols and alkylphenols as the major phenolic compounds. Phenolic acids such as hydroxycinnamic, ferulic, caffeic, and coumaric acids were also observed. Additionally, the *L. minor* extract had a high flavonoid content including several glycosylated forms of kaempferol and quercetin, peonidin, and delphinidin derivatives. The authors attributed the synthesis of ZnO NPs using *L. minor* to the presence of these phenolic metabolites with antioxidant properties, which were able to reduce and stabilise the ZnO NPs. The mechanism they proposed was based on flavonoid and quercetin and is given in Figure 6.

**Figure 7:**
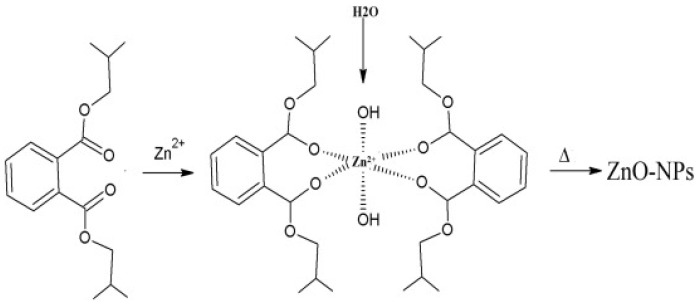
Proposed mechanism of formation of ZnO NPs using *A. italica*. Adapted from Ref. [119].

The proposed mechanism involves the formation of a complex between the Zn (II) ions from the Zn salt precursor and the hydroxyl groups from the quercetin. This is followed by bonding between the OH group of the biomolecule to form Zn(OH)_2_ and decomposition of the Zn(OH)_2_ to the ZnO NPs via calcination. This mechanism was also proposed by Chemingui et al. [118] for the formation of ZnO NPs using *L. nobilis* extract. The authors proposed that the phenolic biomolecules were responsible for the reduction and stabilisation of ZnO NPs via the formation of a complex between the Zn (II) ions and the hydroxyl groups from the phenolic biomolecules. This was followed by hydrolysis of the complex by the hydroxyl from the phenolic biomolecules. Thereafter, the formation of Zn(OH)_2_ and its decomposition via calcination to produce biomolecule-capped ZnO NPs. Additionally, Aziz et al. [119] proposed the same mechanism involving the formation of a complex between Zn (II) ions and diisobutyl phthalate or dibutyl phthalate and hexahydrofarnesyl acetone, which are found in essential oils of the aerial parts of *Anchusa italica* extract. The authors proposed that the complex was formed via the carbonyl group of these molecules, as given in Figure 7.

Moreover, they suggested that the biomolecules were responsible for capping and stabilising the ZnO NPs and preventing agglomeration. The involvement of biomolecules in the synthesis of *A. italica*-mediated ZnO NPs was supported by FTIR analysis of the *A. italica* extract and *A. italica*-mediated ZnO NPs. The authors observed a shift in peak positions and intensity between the FTIR results of *A. italica* extract and *A. italica*-mediated ZnO NPs. The shift in peaks was attributed to the involvement of the functional groups from biomolecules in the *A. italica* extract in the reduction and stabilisation of *A. italica*-mediated ZnO NPs. The absorption bands of *A. italica* extract were 3422, 2943, 1727, 1451, and 1053 cm^−1^, which were shifted to 3352, 2929, 1729, 1411, and 1037 cm^−1^, respectively, which were attributed to contributions from diisobutyl phthalate, hexahydrofarnesyl acetone, tannins, saponins, and flavonoids, with functional groups of carboxylic acid, carbonyl, and alcohols, in the formation and stabilisation of ZnO NPs [119]. Moreover, the presence of biomolecules on the surface of the synthesised ZnO NPs even after calcination proved that the biomolecules are involved in the capping of the synthesised ZnO NPs.

**Figure 8:**
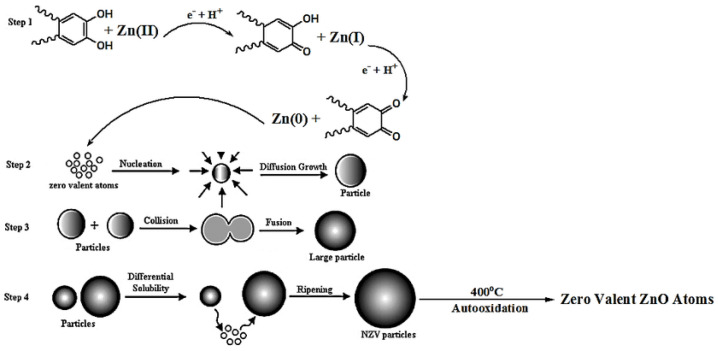
Proposed mechanism of formation of ZnO NPs using *E. petiolate* extract. Adapted from Ref. [120].

A different mechanism of formation of ZnO NPs from plant extracts has been proposed. The mechanism involves the direct reduction of Zn (II) ions from the precursor salt to Zn by the biomolecules from the plant extracts. The reduction is then followed by oxidation of the Zn by oxygen to form the capped ZnO NPs during calcination. Mohammadi et al. [120] proposed a reduction mechanism for the formation of ZnO NPs from *Euphorbia petiolate* (*E. petiolate*) extract. The mechanism involves the reduction of the Zn (II) from the precursor salt by antioxidant phenolic biomolecules from the *E. petiolate* extract to form zero-valent Zn NPs, which is followed by growth of the Zn NPs and finally oxidation to ZnO NPs via calcination, as shown in Figure 8 below.

The same mechanism was proposed by Bhuyan and associates [121] for ZnO NPs synthesised using *A. indica* extract. The authors proposed that the aldehyde groups from the biomolecules present in the *A. indica* extract were responsible for the reduction of Zn (II) ions to ZnO NPs. The authors also mentioned the presence of terpenoids, flavanones, and reducing sugars as some of the major constituents of *A. indica* which are involved in the stabilisation of the synthesised ZnO NPs. The stabilisation of ZnO NPs is evident from the presence of several functional groups reported from the FTIR spectrum of *A. indica*-mediated ZnO NPs. The authors observed bands at 2987 cm^−1^ and 3197 cm^−1^, which were attributed to linkages between amino acids from protein residues, stretching vibrations of amide II, and atmospheric CO_2_. The bands at 1081 cm^−1^ and 1038 cm^−1^ were attributed to C–N stretching vibrations of aliphatic, aromatic amides and aliphatic amines, alcohol and phenolic groups, and stretching vibrations of secondary amines [121]. The presence of these functional groups from the biomolecules in the *A. indica* on the surface of the *A. indica*-mediated ZnO NPs supports the capping of ZnO NPs using plant extracts.

The two proposed mechanisms of formation of ZnO NPs using plant extracts can be summarised using Equations (3)–(6). The mechanism of formation involving the formation of the Zn–biomolecule complex (Equations (3) and (4)) is more plausible and has been proposed by several researchers compared to the mechanism of formation involving direct reduction (Equations (5) and (6)). However, it should be noted that the mechanism of formation of ZnO NPs using plant extracts is not fully understood at the present moment. This is due to the presence of a large variety of biomolecules in the plant extracts as more than one type of biomolecule may be involved in the reduction and stabilisation of the ZnO NPs. Hence, research efforts should also focus on investigating the mechanism of formation of ZnO NPs using plant extracts to best understand the process and establish large-scale production.

(3) $$Zn^{2+}+Biomolecule\to Zn-Biomolecule\left(complex\right)$$

(4) $$Zn-Biomolecule\left(complex\right)\overset{∆}{\to }ZnONPs$$

(5) $$Zn^{2+}+Biomolecule\to Zn+Biomolecule\;(oxidised)$$

(6) $$Zn+O_{2}\left(air\right)\overset{∆}{\to }ZnONPs$$

## 4. Applications

### 4.1. Photocatalysis

#### 4.1.1. Dyes and Wastewater Treatment Technologies

**Figure 9:**
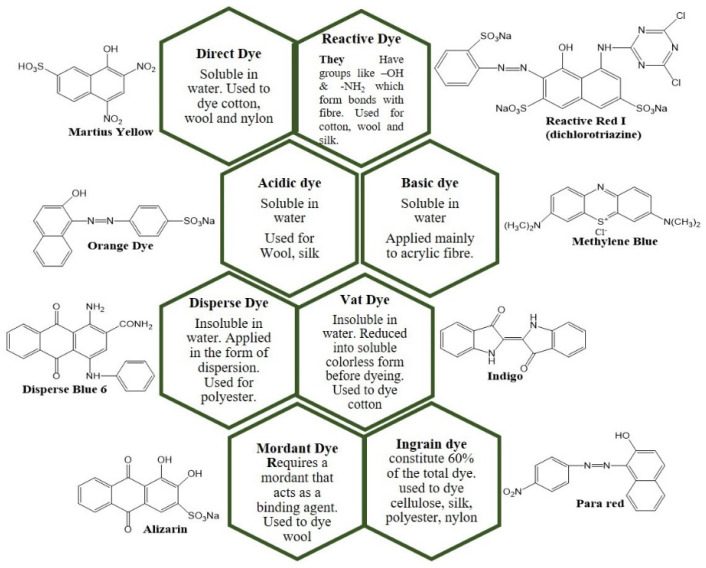
Dye classification. Adapted from Ref. [122].

Dyes are defined as coloured organic substances made up of chromophores and their fixed property of acidic or basic groups that are capable of imparting colour to substrates [122]. Dyes can be categorised as shown in Figure 9.

The release of untreated or inadequately treated industrial wastewater into waterbodies is detrimental to the environment, aquatic life, and human health. This is because dyes are highly carcinogenic and mutagenic, toxic, and highly resistant to biodegradation. Moreover, they can degrade the aesthetic quality of waterbodies, which increases the biochemical and chemical oxygen demand, thus limiting photosynthesis and hence the growth of aquatic life. There is a need to adequately treat industrial wastewater due to the global decline in freshwater resources. This is due to a rapid increase in global population and industrialisation, which has put pressure on freshwater resources. The removal of dyes from industrial wastewater is challenging since organic dyes are resistant to heat, light, oxidation, and biodegradation. This makes conventional wastewater treatment methods such as aerobic and anaerobic treatment inadequate in the removal of the dyes from wastewater. Thus, other wastewater treatment methods have been developed for the removal of dyes from industrial effluent.

Due to the increased use of dyes by industries and the toxic nature of dyes to aquatic life and human health, there is a need to develop effective industrial effluent treatment techniques. Therefore, various effluent treatment techniques have been assessed for the removal of dyes from industrial effluents. There have been several dye removal technologies that have been proposed and studied and are currently in use. Conventional methods such as adsorption, coagulation–flocculation, reverse osmosis, ultrafiltration, and ion exchange have been utilised to treat dye effluents. Dye removal technologies such as coagulation–flocculation, and adsorption generally transfer concentrated pollutants without degradation from one phase to another [123]. Physical methods such as reverse osmosis, ion exchange, and filtration (e.g., ultrafiltration, microfiltration, and nanofiltration) that are currently utilised for the treatment of dye effluents have been associated with various problems such as sludge generation, membrane fouling, disposal issues, handling, and other technical issues [124]. Biological methods which utilise microorganisms and enzymes are an environmentally friendly approach that is used for the treatment of dye effluents; however, the processes are time-consuming and ineffective at treating some dyes, which limits their applications [125]. Chemical methods including oxidation, electrochemical, photolysis, and coagulation–flocculation have been reported to exhibit better dye removal efficiencies on a range of dyes, with removal efficiencies around 88–99%. These methods have been reported to be relatively more costly compared to the physical and biological methods [126]. Additionally, methods such as sedimentation, filtration, chemical, and membrane technologies are hampered by high operation costs and high maintenance costs [127].

Due to the shortfalls of the methods mentioned above, research has focused on heterogeneous photocatalysis, which is an AOP. Photocatalysis has garnered more interest, and this is owing to its non-toxic nature, relative cost-effectiveness, and non-energy intensiveness since operations can take place in ambient conditions. Moreover, there are no secondary pollution concerns because the technique is capable of decomposing organic dyes into harmless compounds [128,129]. The method involves using photocatalysts to generate charge carriers via the absorption of energy that is equal to or greater than the bandgap energy of the photocatalyst. The charge carriers that are generated migrate to the surface where redox reactions take place or recombine, which is termed recombination. ZnO NPs have been utilised extensively for the photocatalytic degradation of dyes due to their properties such as having a low cost, high photostability, high chemical stability, high photoactivity in the UV region, high biological stability, and environmental friendliness [130].

#### 4.1.2. Photocatalytic Mechanism of Zn NPs

**Figure 10:**
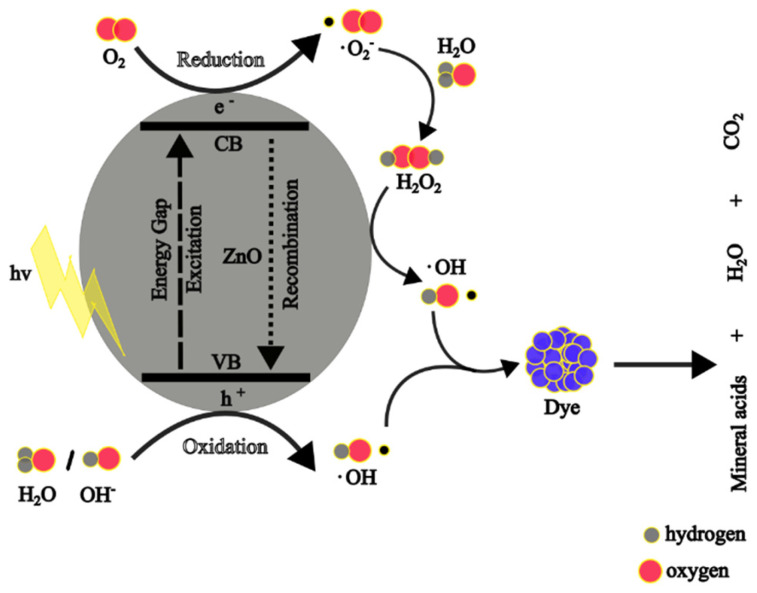
Schematic diagram of the photocatalytic degradation mechanism of dyes using ZnO NPs.

When ZnO NPs are irradiated with energy greater or equal to their bandgap energy, the free electrons (e^−^) in the valence band (VB) absorb the energy and become excited to the free conduction band (CB). This leaves holes (h^+^) in the VB, and the electrons and holes (charge carriers) migrate to the surface of the ZnO NPs. At the surface, the holes react with the water and hydroxyl ion to form hydroxyl (·OH) radicals, whereas the electrons react with the adsorbed oxygen to produce superoxide anion (·O_2_^−^) radicals, which will result in the formation of hydrogen peroxide. The hydrogen peroxide reacts further with ·O_2_^−^ radicals to produce more ·OH radicals. The ·OH radicals are potent ROS and react with the adsorbed organic dyes on the surface of the ZnO NPs, resulting in less harmful products: water and carbon dioxide. The photocatalytic reactions are given from Equations (7)–(16) [90,131], whereas Figure 10 shows a schematic diagram of the photocatalytic degradation mechanism of dyes using ZnO NPs.

(7) $$ZnO\overset{hv}{\to }ZnO\;(h^{+})+(e^{-})$$

(8) $${ZnO\;(h}^{+})+H_{2}O\to {ZnO+H}^{+}+\cdot OH$$

(9) $${ZnO\;(h}^{+})+OH^{-}\to ZnO+\cdot OH$$

(10) $$ZnO\left(e^{-}\right)+O_{2}\to ZnO+\cdot O_{2}^{-}$$

(11) $$\cdot O_{2}^{-}+H^{+}\to \cdot {HO}_{2}$$

(12) $$\cdot {HO}_{2}+\cdot {HO}_{2}\to H_{2}O_{2}+O_{2}$$

(13) $$ZnO\left(e^{-}\right)+H_{2}O_{2}\to \cdot OH+OH^{-}$$

(14) $$H_{2}O_{2}+\cdot O_{2}^{-}\to \cdot OH+OH^{-}+O_{2}$$

(15) $$H_{2}O_{2}+hv\to 2\cdot OH$$

(16) $$\cdot OH+organic\;dyes\to mineral\;acids+CO_{2}+H_{2}O$$

The photocatalytic activity can be assessed using the percentage of degradation (efficiency), which indicates the degree of dye decomposition at any given time. It is achieved by measuring the maximum absorbance or concentration of the dye at a given time and can be represented using Equation (17).

(17) $$\%\;degradation\;efficiency=\frac{A_{0}-A_{t}}{A_{0}}\times 100=\frac{C_{0}-C_{t}}{C_{0}}\times 100$$

where *A*_0_ is the initial absorbance of the dye before photodegradation and *A_t_* is the absorbance of the dye at time *t*, while *C*_0_ is the initial dye concentration and *C_t_* is the dye concentration at time t.

Kinetics models are used to describe and predict the mechanism and performance of photocatalysts during the degradation process. The most commonly used kinetics model in heterogeneous photocatalytic degradation of dyes is the Langmuir–Hinshelwood (L-H), which follows the pseudo-first-order model [132]. The L-H model is described using Equations (18)–(20) [133].

According to the L-H model, when the initial dye concentration is very small, then the following rate equation is used:

(18) $$r=-\frac{dC}{dt}=k_{r}q_{x}\frac{k_{r}KC}{1-KC}$$

where the degradation rate (*r*) is proportional to the t surface coverage of catalyst (*q_x_*) covered by the dye, *k_r_* is the reaction constant, *C* is the concentration of dye at time *t*, and *K* is the adsorption constant of the dye molecule at the surface of the catalyst.

Equation (18) can be rewritten as follows:

(19) $$dt=\frac{1+kC}{k_{r}KC}dC$$

At a low *C*_0_ initial dye concentration, the second part of Equation (19) is negligible; therefore, Equation (19) will become the following:

(20) $$In\left(\frac{C_{0}}{C_{t}}\right)=k_{1}t$$

Plotting a graph of *In*(*C*_0_*/C_t_*) vs. *t* gives the pseudo-first-order constant, *k*_1_*t*.

#### 4.1.3. Photocatalysis Activity of Plant-Mediated ZnO NPs

The particle size of nano-photocatalysts is one of the major factors that influence the photocatalytic activity of NPs. When the particle size of a nano-photocatalyst is small, there is a high surface area-to-volume ratio, which means increased active sites for the adsorption of surface oxygen and dye molecules, thus leading to better photoactivity [134]. The bandgap energy of a nano-photocatalyst is also crucial in their photocatalytic degradation activities. When the bandgap energy of a nano-photocatalyst is small, it means less energy is required to create charge carriers, and it enables them to absorb a greater portion of the electromagnetic radiation, which promotes the generation of the hydroxyl radicals and leads to enhanced degradation. However, a balance is needed because when the bandgap energy is too narrow, recombination of created charge carriers occurs, which reduces the generation of the hydroxyl radicals, resulting in decreased photodegradation [135]. Additionally, the shape of NPs has been reported to influence the photoactivity of the nano-photocatalysts.

Waseem et al. [15] studied the photocatalytic degradation of Methylene blue (MB) dye using *Citrus jambhiri lushi* (*C. jambhiri lushi*) leaf-mediated ZnO NPs under UV light irradiation for 120 min. The authors reported that the highest degradation efficiency was exhibited by the *C. jambhiri lushi*-mediated ZnO NPs with a smaller bandgap compared to those with a larger bandgap. Maro and associates [136] conducted photodegradation studies of MB, MO, and Rhodamine B (RhB) dyes using *Peumus boldus* (*P. boldus*)-mediated ZnO NPs with varying bandgaps. The degradation efficiencies increased with a decrease in the bandgap of the synthesised ZnO NPs. The authors revealed that *P. boldus*-mediated ZnO NPs with the smallest bandgap (2.80 eV) had the highest degradation efficiency against MO (92% in 180 min) compared to 42 and 47% for *P. boldus*-mediated with bandgaps of 3.00 and 3.10 eV, respectively. The same trend was reported for the degradation efficiency of *P. boldus*-mediated against MB and RhB dyes. This is because lower bandgaps allow the generation of more charge carriers, which are responsible for the production of hydroxyl radicals that are responsible for the degradation of dyes, resulting in better photoactivity.

A study compared the photocatalytic activity of nano-flowered ZnO NPs prepared with *Withania coagulans* (*W. coagulans*) fruit extract and chemical methods against MB dye under sunlight irradiation in 120 min. The authors reported that the degradation efficiency of *W. coagulans*-mediated ZnO NPs (90%) was higher than the chemical-mediated ZnO (78%). The difference was attributed to the smaller crystallite size and reduced bandgap of *W. coagulans*-mediated ZnO NPs (25 nm and 3.35 eV, respectively) compared to chemical-mediated ZnO NPs (30 nm and 3.44 eV, respectively) [137]. Bekele and co-workers [138] compared the photocatalytic activity of ZnO NPs prepared using varying ratios of *Acanthus sennii* leaf extract to Zn salt precursor volume (1:1, 2:3, and 3:2) against Acid orange 88 dye in 120 min under UV light irradiation. Based on the XRD results, the authors revealed that the 1:1-mediated ZnO NPs had the smallest crystallite (19.55 nm), which resulted in their better degradation efficiency (62.6%) despite having a higher bandgap (3.31 eV) than 2:3- and 3:2-mediated ZnO NPs with 3.28 and 3.25 eV, respectively. The efficiencies of 2:3- and 3:2-mediated with crystallite sizes of 34.19 and 23.07 nm in degradation of Acid orange dye were reported to be 57.25 and 34.45%, respectively. The difference in photodegradation performances was attributed to the greater surface area-to-volume ratio due to the smaller size of 1:1 ZnO NPs and the presence of biomolecules on the surface.

In another study, Rafique et al. [139] investigated the photoactivity of ZnO NPs synthesised with varying *Citrus reticulata* (*C. reticulata*) volumes (10, 20, and 30 mL) against RhB dye under UV light irradiation in 160 min. The study reported that the photocatalytic activity increased (60, 75.78, and 85.97%, respectively) with an increase in *C. reticulata* volume owing to the decrease in particle sizes (40–80, 30–70, and 20–30 nm, respectively). Additionally, the differences in the shape of the synthesised ZnO NPs could have contributed to the differences in degradation efficiencies as the shapes of *C. reticulata*-mediated ZnO NPs were observed to be irregular, spherical/flowers, and flowers, respectively.

Shanavas et al. [140] investigated the difference in photocatalytic activity of plant-mediated ZnO NPs synthesised using different plants against RhB dye under UV exposure for 180 min. The authors synthesised ZnO NPs using *Artabotrys hexapetalu* (*A. hexapetalu*) and *Bambusa vulgaris* (*B. vulgaris*) leaf extracts. Based on the XRD results and bandgap energy calculations, the crystallite sizes were 29 and 19 nm, respectively, and the bandgaps were 3.06 and 3.12 eV, respectively. *A. hexapetalu*-mediated ZnO NPs exhibited 92% and 1.4 × 10^−2^ min^−1^ degradation efficiency and rate constant, respectively, which was slightly above *B. vulgaris*-mediated ZnO NPs, with degradation efficiency and rate constant of 88% and 1.21 × 10^−2^ min^−1^, respectively. The authors attributed the slight difference in degradation performance to biomolecules on the surface of the *A. hexapetalu*-mediated ZnO NPs that can aid in dye molecule adsorption, thus bringing the dye molecules to the catalytic surface [140]. Additionally, the slight difference in their photoactivity could also be related to the smaller bandgap energy of *A. hexapetalu*-mediated ZnO NPs compared to *B. vulgaris*-mediated ZnO NPs. Therefore, more studies are needed to understand the photocatalytic activity of NPs.

Alharthi et al. [141] investigated the photocatalytic degradation of MB dye using *Salvadora Persica*-mediated ZnO NPs with different morphologies. The *Salvadora Persica*-mediated ZnO NPs with some nanorods exhibited significantly better degradation efficiency (95% in 150 min) than the *Salvadora Persica*-mediated ZnO NPs with some spherical particles (75 in 150 min). The authors attributed this difference to the varying morphologies of the synthesised NPs, the presence of the nanocavities and the nanorods offered a higher surface area for degradation to take place. In another study, the photodegradation of MB dye was carried out using *Heliotropium indicum*-mediated ZnO NPs of different morphologies. The authors reported that the spherical ZnO NPs exhibited a higher degradation efficiency than flower-shaped ZnO NPs under visible light irradiation [142].

All these observations validate the influence of properties of plant-mediated ZnO NPs on degradation efficiency. However, it should be noted that photocatalytic degradation is a complex process and does not only depend on the properties like size, bandgap, and shape of the NPs. There are other factors such as surface defects and crystallinity of the plant-mediated ZnO NPs that also come into play. Therefore, a better understanding of the process will help synthesise plant-mediated ZnO NPs that are tailored for the effective degradation of dyes. For example, the bandgap of the plant-mediated ZnO NPs can be tailored by doping and coupling the NPs with semiconductors [143].

In addition to the properties of plant-mediated ZnO NPs influencing the photodegradation processes, reaction parameters are also crucial during photodegradation, and there is a need to optimise these parameters for effective photocatalytic activity. Parameters, such as initial pH, catalyst dosage, and initial dye concentration are some of the parameters that have been studied during photodegradation processes. The photodegradation efficiency has been reported to increase with an increase in catalyst dosage. This is due to the presence of a greater number of active sites for the generation of ROS, thereby increasing the decomposition of the dyes until an optimal dosage is reached. Increasing the catalyst dosage beyond the optimum threshold is associated with a decrease in the photodegradation efficiency. This may be due to particle agglomeration, which reduces the uniformity of the suspension and light scattering as a result of solution turbidity, thereby resulting in reduced active sites [144].

In heterogeneous catalysis, when the initial concentration of the dye is increased beyond the optimum concentration while other parameters remain constant, the photodegradation efficiency is reduced. This can be attributed to the decrease in the ratio of active sites to dye molecules and the ratio of hydroxyl radicals to dye molecules. The adsorption of hydroxyl radicals will decrease, while the adsorption of the dye molecules increases, which in turn decreases the rate of formation of the hydroxyl radicals. Additionally, there is a reduction in photons of light reaching the ZnO NP surface due to increased absorption of light by the dye concentration as the concentration increases, resulting in a reduction in degradation efficiency [145].

pH plays an important role in photodegradation performance. It influences several factors during the degradation process which include the surface charge of ZnO, the charge of the dye molecules, the concentration of the hydroxyl radicals, and the adsorption of the dye molecules on the ZnO NP surface. The surface charge of ZnO NPs can be explained using the point of zero charge (pH_pcz_), which is the pH where the concentration of protonated and deprotonated groups is the same. The pH_pcz_ of ZnO is estimated to be 9, and at a pH lower than 9, the surface is protonated (positively charged), whereas, at a pH greater than 9, the surface is deprotonated (negatively charged) as a result of the adsorbed hydroxyl ions. A high concentration of hydroxyl ions on the surface of ZnO and in solution may promote the formation of the hydroxyl radical responsible for the degradation of dyes, thereby increasing the degradation efficiency [146].

The effect of pH on the photocatalytic degradation process can also be explained by electrostatic interactions between charged surfaces, ions, and molecules. In some instances, the electrostatic repulsion between the negatively charged ZnO and hydroxyl ions can reduce the degradation efficiency in alkaline conditions. Additionally, the charge of the dye molecules also affects their interaction with the charged surface of ZnO, thus affecting the adsorption process. Under acidic conditions, anionic dyes are strongly adsorbed, while cationic dyes are strongly adsorbed under basic conditions. It should be noted that ZnO is an amphoteric metal oxide that dissolves at pH < 3 and pH > 11, which affects the degradation efficiency [147]. Considering these factors, it is important to optimise the pH of the photodegradation process to maximise the degradation activity.

The influence of photodegradation parameters was investigated by Nethravathi et al. [148] for the degradation of MB dye by *Garcinia xanthochymus*-mediated ZnO NPs. The process involved varying the initial dye concentrations (5 to 20 ppm), pH of the reaction mixture (2 to 12), and *G. xanthochymus*-mediated ZnO NP dosage (50 to 200 mg). The optimum conditions were reported to be 5 ppm, 200 mg, and pH 4, respectively. The researchers reported that the degradation increased with an increase in pH; however, a further increase beyond pH 4 resulted in a decrease in efficiency. They attributed this observation to the presence of positively charged ZnO NPs at acidic pH, which facilitates the attraction of the negatively charged MB dye molecules, thereby increasing efficiency. As the pH is increased further, the ZnO NPs become negatively charged owing to the adsorption of hydroxyl ions. The effect of nano-photocatalyst dosage was studied using *Ficus racemose* (*F. racemose*)-mediated ZnO NPs for the photodegradation of RhB dye. The degradation efficiency was revealed to increase with an increase in *F. racemose*-mediated ZnO NPs loading from 20 to 60 mg. However, further increase of the *F. racemose*-mediated ZnO NPs loading beyond 60 mg, resulted in a reduction in the degradation efficiency. The authors suggested 60 mg as the optimum dosage for the degradation of RhB dye using *F. racemose*-mediated ZnO NPs [149]. In another study, the effect of photodegradation parameters in the photodegradation of Congo red (CR) and Malachite green (MG) using *Morinda umbellate* (*M. umbellate*)-mediated ZnO NPs was investigated. The authors investigated the effect of initial dye concentration from 10 to 50 ppm and reported that at low dye concentrations, the rate was proportional to the dye concentrations. However, at higher dye concentrations, the degradation efficiency decreased with an increase in the initial dye concentration. The authors suggested that the decrease in efficiencies for both dyes at high initial dye concentrations was a result of the reduced number of active NPs due to the increased adsorption of dyes, limited light penetration, and reduced ROS generation. The optimum parameters for degradation of CR dye using *M. umbellate*-mediated ZnO NPs were deduced as 10 ppm CR concentration, 0.125 g *M. umbellate*-mediated ZnO NPs, and pH 6, whereas the optimum parameters for MG were 20 ppm dye concentration and 0.1 g *M. umbellate*-mediated ZnO NPs. However, the degradation efficiency of the cationic MG increased in all the pHs (3–10) [150].

All the above observations suggest that plant-mediated ZnO NPs have potential as alternatives to chemical and physically synthesised ZnO NPs for photocatalytic degradation of organic dyes. It should however be noted that the properties of plant-mediated ZnO NPs such as particle size, optical bandgap, and shape influence their photoactivity. These properties can be controlled and manipulated during the synthesis of the plant-mediated ZnO NPs, leading to better photoactivity. The photocatalytic degradation process is very complex in nature and can be influenced by several factors at the same time. Therefore, a better understanding of the photodegradation process will lead to the development of high-performing photocatalysts. 

The reusability of nano-photocatalysts is an important factor in photodegradation studies due to its correlation with the cost-effectiveness and sustainability of the process, which is important in large-scale applications. Reusability studies conducted on *Borreria hispida*-mediated ZnO NPs in the degradation of Malachite Red (MR) dye revealed the photostability of plant-mediated ZnO NPs, evident from the reduction in efficiency from 94.24 to 84.8% after five cycles [151]. Bopape et al. [152] reported a significant reduction in the degradation efficiency of MB dye from 81 to 37% after four cycles of degradation using *Commelina benghalensis*-mediated ZnO NPs under UV light irradiation. They attributed this reduction to the loss of catalyst, catalyst poisoning, catalyst coverage, etc. Similarly, the photodegradation of Alizarin Yellow R dye using *Serratula coronate*-mediated ZnO NPs as a photocatalyst reduced from 98.96 to 55% after six degradation cycles. The authors suggested that the reduction in efficiency was a result of the deactivation of the active sites [153].

Several studies have proposed and explored remedies to improve the reusability of nano-photocatalysts during photodegradation processes. Some of these include immobilising the NPs on supports to improve their reusability to facilitate easy separation of the nano-photocatalysts from the reaction media. NPs can be immobilised on different support materials such as activated carbon, glass, cellulose, plant fibres, metals, clay, zeolites, polymers, hydrogels, etc. [154]. To the best of our knowledge, there are no reports in the literature about the embedding of plant-mediated ZnO NPs on supports to facilitate ease of recovering the NPs in large-scale degradation settings. Therefore, some research efforts can be focused on exploring this avenue.

Herein, we discuss some of the support materials that have been used with chemical-mediated ZnO NPs. Hydrogels made from carboxymethyl cellulose (CMC) and sodium alginate (SA) have been used as supports of NPs for the photodegradation of pollutants. CMC and SA are naturally polysaccharides that are used to produce hydrogels through hydrogen bonding that are eco-friendly, with good mechanical, thermal, and chemical stability. Hydrogels have high adsorption capacity and can adsorb dye molecules, which facilitates the degradation of the dye molecules by the embedded ZnO NPs, thereby improving photodegradation activity. Ramadhani and Helmiyati [155] synthesised ZnO NPs and embedded the NPs in a CMC–alginate hydrogel that was crosslinked using Ba (II) ions. They reported that the hydrogel–ZnO composite exhibited a remarkable degradation efficiency of 90.12% in 110 min for the degradation of CR dye under visible light irradiation. The reusability experiments revealed that the efficiency decreased from 90.12 to 69.63% in three cycles. ZnO NPs have also been embedded in Kaolinite (KL), a highly abundant material that has a rigid structure and high chemical stability. The ZnO/KL composite was utilised for the photodegradation of 2-chlorophenol, and the authors reported complete degradation of the 2-chlorophenol in 180 min. The reusability tests revealed that the ZnO/KL composite was easy to recover and photostable as evidenced by the excellent stability of the degradation efficiency after five consecutive cycles [156].

Bel Hadjltaief et al. [157] immobilised ZnO NPs on Tunisian natural clay (TNC) support and utilised the composite for photodegradation of MG and CR dyes under solar and UV irradiation. The ZnO-TNC composite was reported to exhibit remarkable degradation efficiencies for both dyes under solar compared to UV irradiation. The reusability test revealed that the efficiencies were stable up to the fourth cycle for both dyes, and a slight reduction was observed on the fifth cycle. These findings demonstrate that immobilising ZnO NPs on supports can enhance their photocatalytic activity and also improve their photostability, thus promoting their reusability potential. Additionally, it provides a means for easy separation and recovery of NPs that is relatively cost-effective. Therefore, there is a need to investigate the immobilisation of plant-mediated ZnO NPs on supports.

### 4.2. Antibacterial

Antibiotics are “magic bullets” of the 20th century that are used as antibacterial agents to combat infections caused by bacteria. Even though antibiotics have helped fight infections for years, their use and misuse have given rise to antibiotic-resistant infections. The increase in antibiotic resistance in bacteria is a global threat to both humans and animals, especially in developing countries, as it results in hundreds of thousands of deaths per year [158]. Therefore, there is a need to develop antibacterial agents that are affordable, safe, and capable of combatting antibiotic-resistant infections. Several strategies have been proposed to overcome antibiotic-resistant infections and amongst them, the use of NPs as non-traditional antibacterial agents has been investigated. Plant-mediated ZnO NPs have been investigated as potential antibacterial agents, and they have shown exceptional antibacterial activity.

Several methods evaluating the antibacterial activity of ZnO NPs in vitro have been reported and the most common methods include agar dilution, broth dilution, and disc diffusion. The disc diffusion or agar well diffusion method involves inoculating agar plates with a standardised concentration of bacteria. This is followed by adding filter paper or making wells containing the desired concentration of the antibacterial agent on the agar plate and incubating the Petri dish under suitable conditions. The antibacterial agents will diffuse through the agar and inhibit bacterial growth. This can be observed as discs called inhibition zones which can be measured, and the antibacterial activity can be given as inhibition zone diameter (ZOI) expressed in millimetres (mm) [159]. The agar dilution and broth dilution methods are used to quantitatively measure the in vitro antibacterial activity. Generally, the agar dilution method involves making varying concentrations of antibacterial agents using two-fold serial dilution and adding them to the agar medium (agar dilution) or liquid medium (broth) in plates or tubes. The inoculum is then incubated at suitable conditions and the antibacterial activity is given as the minimum inhibition concentration (MIC), which is the smallest concentration of the antimicrobial agent that completely inhibits bacteria growth [160].

#### 4.2.1. Antibacterial Mechanism of ZnO NPs

To the best of our knowledge, five antibacterial mechanisms of ZnO NPs have been proposed, which are (1) ROS generation through photocatalytic activity; (2) induction of oxidative stress; (3) attachment of the ZnO NPs to the bacteria cell walls; (4); release of Zn (II) ions; and (5) genotoxicity [161]. The most accepted antibacterial mechanisms involve the generation of ROS in the presence of light. The mechanism is similar to the ROS generation for photocatalysis discussed in the sections above. The hydroxyl and superoxide radicals interact with the surface of the cell membrane, thereby destroying the cell membrane, while hydrogen peroxide enters the cells, causing damage and destruction of DNA and proteins, which leads to the death of the bacteria [162]. However, a study by Jiang et al. [163] reported that the hydroxyl radicals rather than the superoxide and the hydrogen peroxide are responsible for the death of bacteria cells due to their higher reactivity compared to the superoxides. Other studies have reported the induction of oxidative stress through the generation of ROS in the absence of light. Defects can occur on the surface of ZnO NPs, and these defects can be sites for generating ROS, which is responsible for the destruction of bacteria cell membranes. However, this mechanism has been reported to be less efficient compared to the photocatalytic generation of ROS [164].

**Figure 11:**
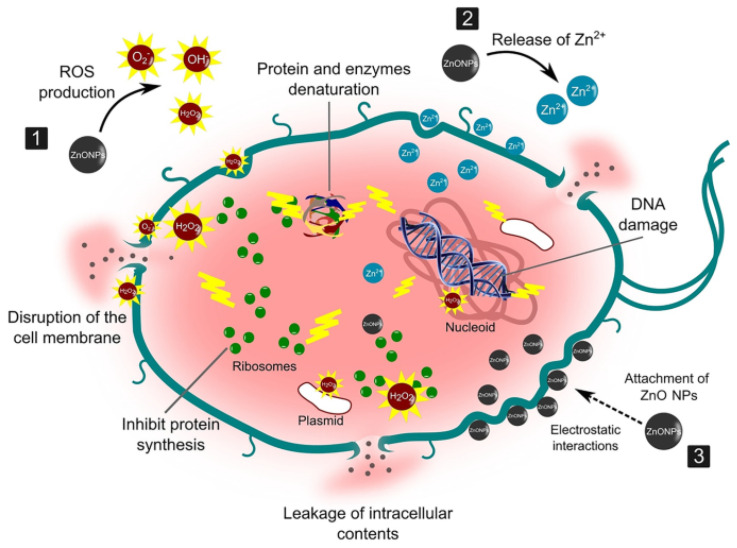
ZnO NPs’ antibacterial mechanisms. Adapted from Ref. [165].

The third mechanism that has been proposed involves the interaction of ZnO NPs with the bacteria, destroying the surface of the bacteria. The interaction occurs due to physical interaction such as electrostatic interactions between the positively charged ZnO NPs and the negatively charged bacteria cells. The interaction of ZnO NPs with the surface of the cell membrane also increases its permeability, thus allowing ZnO NPs to enter the cell. Moreover, particles that are less than 10 nm can also penetrate the cell. Inside, the ZnO NPs inhibit metabolic reactions, resulting in cell death [165]. Another mechanism that has been proposed involves the penetration of Zn (II) ions from the dissolution of ZnO NPs. The Zn (II) ions can be adsorbed on the surface of the bacteria and interact with the surface, resulting in increased permeability. This allows the Zn (II) ions to enter the cell and inhibit cell activities such as glycolysis, proton transport system, and acid tolerance, leading to cell death [166]. The final mechanism that has been proposed is genotoxicity, and it occurs through the interaction of the metal ions from the NPs with the phosphoric residues and proteins from the bacteria DNA. This interaction can result in the inhibition of DNA processes such as cell replication and division [161]. Figure 11 shows some of the proposed antibacterial mechanisms using ZnO NPs.

The mechanism of antibacterial activity of NPs is not fully understood at the present moment. Therefore, further studies are needed to understand the mechanism, which will result in the realisation of the full potential of the NPs as antibacterial agents. Understanding the mechanism of antibacterial action of plant-mediated ZnO NPs is very important for public health, environmental safety, and industrial advancement. Understanding the mechanism of antibacterial action of plant-mediated ZnO NPs can help develop more effective antibacterial agents that can combat antibiotic resistance and design better antibacterial coatings to help fight infections to safeguard public health [167]. Additionally, it can result in the development of more effective antibacterial agents for use in the food and food packaging industry to help prolong the shelf-life of food and prevent bacterial contamination. Foodborne illness as a result of bacterial contamination is a major threat to global health [168,169].

#### 4.2.2. Antibacterial Activity of Plant-Mediated ZnO NPs

The antibacterial activity of plant-mediated ZnO NPs has been extensively reported in the literature owing to their biocompatibility and non-toxicity. Additionally, it has been reported that plant-mediated ZnO NPs exhibit a higher degree of antibacterial activity compared to chemical-mediated ZnO NPs. This can be attributed to synergic efforts from the ZnO NPs and the biomolecule capping agents on the surface of the NPs. For example, Ravbar et al. [170] conducted a study that revealed that plant-mediated ZnO NPs have better antibacterial activity compared to chemical-mediated ZnO NPs. The antibacterial activity of the Japanese knotweed root-mediated ZnO NPs was significantly greater compared to chemical-mediated ZnO NPs against *Campylobacter jejuni* and *S. aureus* bacteria. This was evident from the lower MICs (62.5 and 500 mg/L, respectively) for biosynthesised ZnO NPs compared to chemical-mediated ZnO NPs (500 and 2000 mg/L). Similar findings were reported by Muthuvel and co-workers [108] using *S. nigrum*-mediated ZnO NPs and chemical-mediated ZnO NPs against five bacteria (*Bacillus subtilis* (*B. subtilis*), *Staphylococcus saprophyticus* (*S. saprophyticus*), *Eschechiria coli* (*E. coli*), and *Pseudomonas aeruginosa* (*P. aeruginosa*)). The authors observed that the ZOIs at a 50 µL ZnO NP dosage were 11, 8, 12, and 9 mm, respectively, for the biosynthesised ZnO NPs, which were significantly higher than chemical-mediated ZnO NPs at the same concentration (no activity, 1, 2, and 1 mm, respectively). 

In another study, Mahalakshmi et al. [171] conducted a comparative investigation of the antibacterial activity of *Sesbania grandiflora* (*S. grandiflora*)-mediated ZnO NPs and chemical-mediated ZnO NPs against *S. aureus* and *P. aeruginosa*. The authors reported better antibacterial performance of *S. grandiflora*-mediated ZnO NPs compared to chemical-mediated ZnO NPs. In another study, the antibacterial activity of *A. indica*-mediated ZnO NPs was compared to chemical-mediated ZnO NPs. *A. indica*-mediated ZnO NPs recorded significantly greater antibacterial activity against five bacteria compared to the chemical-mediated ZnO NPs. The measured ZOIs for *A. indica*-mediated ZnO NPs were 23.3, 22.1, 21.3, 20.0, and 20.6 mm and 14.3, 9.6, 7.6, 9.3, and 9.8 mm for *S. aureus*, *B. subtilis*, *P. aeruginosa*, *Proteus mirabilis* (*P. mirabilis*), and *E. coli*, respectively. The greater performance of *A. indica*-mediated ZnO NPs compared to chemical-mediated ZnO NPs was attributed to the smaller particle size of *A. indica*-mediated ZnO NPs, which resulted in higher surface area and better interaction of the NPs with the bacteria [172]. Additionally, the significantly better antibacterial potency of plant-mediated ZnO NPs against bacteria compared to chemical-mediated ZnO NPs can be attributed to the synergic efforts of the ZnO NPs and the biomolecules capped on the surface of the NPs. For example, polyphenols from plants have been reported to possess antibacterial properties, and they interact with their proteins, cell walls, damage to DNA, etc., ultimately leading to the destruction of the bacteria [173].

Bacteria can be divided into two major groups, Gram-positive (G+) and Gram-negative (G−) bacteria, based on the structure of their cell walls, which provide support and help protect the bacteria from physical stress. The G− bacteria are surrounded by an outer membrane made up of two lipid bilayers that are separated by a thin peptidoglycan layer of about 3–8 nm in thickness, whereas G+ bacteria consist of a thicker peptidoglycan layer that has a thickness of about 20–30 nm but lacks the lipid bilayer [174]. Due to these structural differences, some studies have reported a difference in the antibacterial activity of ZnO NPs against the G+ and G− bacteria. For instance, Imade et al. [175] investigated the antibacterial activity of plantain peel-mediated ZnO NPs against two G+ and two G− bacteria. Significantly higher activity was reported for G+ *S. aureus* bacteria compared to the G− *Salmonella enterica*, evident from the ZOIs of 27.67 mm and 10.67 mm, respectively. The authors attributed this difference to the presence of the lipid bilayer in G− bacteria that is harder for the ZnO NPs to penetrate.

However, other studies have reported a higher degree of antibacterial efficacy of plant-mediated ZnO NPs against G− bacteria than G+. Rambabu and co-workers [176] investigated the antibacterial activity of *Phoenix dactylifera*-mediated ZnO NPs against two G+ and two G− bacteria. The authors observed that the antibacterial activity of the prepared ZnO NPs at a 100 µg/mL dosage was relatively higher against G− bacteria (*Streptococcus pyogenes*) with a ZOI of 14.5 mm compared to G+ (*Proteus mirabilis*) with a ZOI of 18.7 mm. The authors attributed these observations to a thin peptidoglycan cell wall that is easier to penetrate in G− bacteria. Contrary to these findings, some studies have reported no significant differences in the antibacterial activity of plant-mediated ZnO NPs on both G− and G+ bacteria. Archana et al. [177] studied the antibacterial activity of *Cucumis melo* (*C.melo*)-mediated ZnO NPs against two G− and two G+ bacteria. The authors reported that there was no significant difference in the antibacterial efficacy of *C. melo*-mediated ZnO NPs at a 150 µL dosage for both G+ and G− bacteria as supported by the ZOIs of 9.50, 9.85, 8.90, and 9.25 mm for G+ (*S. aureus* and *B. subtilis*) and G− (*E. coli*, and *P. aeruginosa*) bacteria.

Similar to the photodegradation of dyes, the antibacterial activity of plant-mediated ZnO NPs can be influenced by the properties of the synthesised ZnO NPs, such as size and morphology. This was demonstrated in a study by Alnehia and associates [178]. The study investigated the antibacterial efficacy of 200 mg/L pomegranate peel-mediated ZnO NPs against *E. coli*. The authors noted that smaller pomegranate peel-mediated ZnO NP particles (18.53 and 20 nm) exhibited better antibacterial efficacy than larger ones (30.34 nm). This was evident from the measured ZOIs of 27, 25, and 17 mm, respectively. Additionally, the authors reported that the pomegranate-mediated ZnO exhibited better efficacy on G+ than G− bacteria. In a similar study, *A. indica*-mediated ZnO NPs of different particle sizes were investigated for antibacterial activity against *S. aureus* and *E. coli*. The particles annealed at 600 °C had a smaller particle size (20 nm) compared to those annealed at 100 and 300 °C (107 and 87 nm, respectively). Therefore, the particles annealed at 600 °C were reported to exhibit significantly greater antibacterial activity than the particles annealed at 100 and 300 °C [113]. Smaller particles exhibit better antibacterial efficacy due to having a larger surface area than larger particles, which aids in the generation of ROS, thereby leading to better antibacterial activity. Additionally, smaller particles can penetrate the bacteria cells, unlike larger particles, and cause oxidative stress, leading to the death of the bacteria cells [179].

In another study, Darvishi and co-workers [180] found that spherical-shaped *Juglans regia* (*J. regia*) leaf-mediated ZnO NPs with particle sizes ranging between 45 and 65 nm had significantly higher antibacterial potency than the flower-shaped ones, with particle sizes ranging between 95 and 150 nm against infection-causing bacteria (*E. coli*, *P. aeruginosa*, and *Acinetobacter baumannii*). The authors attributed this to the greater surface area and presence of a large number of biomolecules on the surface of the smaller particles. Additionally, the spherical-shaped *J. regia*-mediated showed greater antibacterial activity compared to chemical-mediated ZnO NPs and *J. regia* extract. Sharma et al. [94] also studied the influence of shape on the antibacterial activity of aloe vera-mediated ZnO NPs. The authors reported that cuboidal-shaped ZnO NPs exhibited relatively better antibacterial efficacy against *S. aureus*, *B. subtilis*, and *E. coli* bacteria than the spherical and hexagonal-shaped ones. All these findings demonstrate that plant-mediated ZnO NPs possess outstanding antibacterial properties and are effective on different types of bacteria.

## 5. Conclusions and Recommendations

Green synthesis of ZnO NPs using plants is a viable synthesis method that has the potential to replace chemical and physical synthesis methods as it offers a cheaper and environmentally friendly option. Biomolecules present in plant extracts can successfully reduce Zn salt precursors and stabilise ZnO NPs, offering an alternative route to produce ZnO NPs sustainably. The properties of plant-mediated ZnO NPs are dependent on reaction parameters such as pH, reaction temperature, annealing temperature, plant concentration, and Zn salt precursors. Therefore, controlling and optimising these parameters during synthesis is crucial in producing NPs of diverse applications. However, control of properties such as the size and shape of plant-mediated ZnO NPs might be difficult due to the need to optimise several steps of the synthesis process, like pretreatment of the plant, extraction process, and the synthesis of the plant-mediated ZnO NPs. The mechanism of plant-mediated synthesis of ZnO NPs is not fully understood presently; therefore, further studies are required to understand the mechanism as it is crucial in the reproducibility of the synthesis process as well as tailoring the properties of ZnO NPs. Furthermore, research efforts should focus on studies on the reproducibility and scalability of the plant-mediated synthesis processes.

Plant-mediated synthesis has been successfully utilised for the photodegradation of dyes and antibacterial studies. Generally, plant-mediated ZnO NPs exhibited better photoactivity against organic dyes and antibacterial activity than chemical-mediated ZnO NPs. Properties of ZnO NPs such as particle size, optical properties, and morphology can influence the photodegradation of dyes and the antibacterial activity of plant-mediated ZnO NPs. Therefore, tailoring these properties during synthesis is crucial for effective performances in both applications. Moreover, embedding plant-mediated ZnO NPs on supports such as hydrogels can improve their reusability in photodegradation applications. Reusability tests of the plant-mediated ZnO NPs have demonstrated their photostability; however, studies on the immobilisation of the plant-mediated ZnO NPs are needed as embedding NPs promotes easy separation and recovery of photocatalysts.

The significantly better performance of plant-mediated ZnO NPs compared to chemical-mediated ZnO NPs shows that plant-mediated ZnO NPs have the potential to contribute towards fighting antibiotic resistance and applications in industries such as the food and packaging industry. However, there is a need to standardise the plant-mediated synthesis of the ZnO NPs for reproducibility. Additionally, research efforts should focus on understanding the mechanism of antibacterial activity of plant-mediated ZnO NPs as this will help in optimising their synthesis for clinical applications. Studies on understanding the interactions of plant-mediated ZnO NPs with biological systems are also needed to evaluate the toxicity that may result from the interactions between the NPs and biological systems.

## Figures and Tables

**Table 1:** The advantages and disadvantages of green synthesis of ZnO NPs.

Advantages	Disadvantages
Cost-effective	Reproducibility issues
Biocompatible	Relatively less stable NPs
Low energy consumption	Difficult to control morphology
Avoids hazardous chemicals	-
Does not require expensive equipment	-

**Table 2:** ZnO properties.

Property	Value
Appearance	White solid
Odour	Odourless
Molecular weight	81.38 g/mol
Density	5.6 g/cm^3^
Coordination geometry	Tetrahedral
Crystal structure	Hexagonal wurtzite
Melting point	1975 °C (decomposes)
Flash point	1436 °C
Toxicity hazards	None
Refractive index	2.0041
Bandgap	Direct and 3.37 eV at RT
Electrical conductivity	Semiconductor
Hole effective mass	0.59
Electron effective mass	0.24
Exciton binding energy	60 meV
Bohr radius	2.34 nm
Luminescence	Luminescent in UV and visible light

**Table 3:** The advantages and disadvantages of some physical synthesis methods.

Synthesis Method	Advantages	Disadvantages	References
Ball milling	Efficient and ideal for large-scale production.	Lengthy processing times.	[39]
Thermal evaporation	High deposition rates.	Poor coverage due to low vacuum.	[48]
Physical vapour deposition	Simple and relatively low-temperature method.	High production costs and low productivity.	[49]
Molecular beam epitaxy	Produces high-purity materials.	High operational cost and expensive equipment.	[48]
Sputtering	The nature of sputtering gas may affect the properties of the thin films.	High cost of target and low purity.	[47,48]

**Table 4:** Advantages and disadvantages of some chemical synthesis methods.

Synthesis Method	Advantages	Disadvantages	Reference
Sol–gel	Easy and low-temperature synthesis.	Utilises chemical agents and metal alkoxides are expensive	[65]
Hydrothermal	A simple method that produces high-yield and uniform NPs.	Requires an autoclave, and high temperatures. Risk of explosion from the autoclave.	[66]
Chemical reduction	It is cost-effective and has a good production rate.	Utilises hazardous agents and the formation of toxic by-products.	[67]
Chemical vapour deposition	Produces high-purity NPs.	Operates at high temperatures and utilises chemical agents.	[68]
Microemulsion	No energy requirements.	Difficult to scale up and requires large amounts of surfactants.	[65]

**Table 5:** Different morphologies of *P. dactylifera*-mediated ZnO NPs synthesised using different types of Zn salt precursors.

Type of Zn Salt Precursor	Morphology
Zinc chloride anhydrous	Mixture of random, conglomerated, packed, clustered, and rod-shaped NPs with face-centred rhombohedral, spherical, hexagonal, and cubic-like structures
Zinc acetate dihydrate	Needle-shaped nanorods, thinner nanoplates, and flower-like nanolayers
Zinc nitrate hexahydrate	Nanorods, flower-like, nanolayered, and some cubic structures
Zinc sulphate monohydrate	Nanorods, nanoplate flower-like nanolayered, and hollow spherical

**Table 6:** Properties and applications of some plant-mediated ZnO NPs.

Plant	XRD	Particle Size	Shape	Application	Ref.
*M. acuminata*	30 nm 33.3 nm 40.0 nm	-	Leaf-like, triangular-like, triangular with tapered tips	Photocatalysis	[97]
*C. roseus*	Average 36.83 nm	62–94 nm SEM 50–92 nm TEM	Spherical	Antimicrobial	[98]
Cherry	20.18 nm	87.5–116 nm	Spherical	-	[101]
*H. sabdariffa*	8.71 nm 9.05 nm 38.63 nm	5–12 nm 20–40 nm TEM	Spherical	Photocatalysis	[107]
*S. nigrum*	Average 2.07 nm	Average 49 nm DLS	Spherical	Antibacterial Antioxidant Photocatalysis	[108]
*M. acuminata*	Average 14.93 nm	Average 74.19 nm BET	-	-	[92]
*P. oleracea*	25.39 nm 26.04 nm	Average size distribution 70 nm	Spherical and oval	Photocatalysis	[100]
*T. cacao*	-	Average 81 nm TEM	Irregular	Antibacterial Photocatalysis	[105]
*C. halicacabum*	Average 48 nm	Average 48 nm DLS	Hexagonal quartzite	Antibacterial	[107]
*P. dactylifera*	9.3–22.6 nm	3.7–10.2 nm TEM	nanorods, nanoplate flower-like, and hollow spherical; needle-shaped nanorods, thinner nanoplate, and flower-like; nanorods, flower-like, and some cubic; clustered and rod-shaped NPs with face-centred rhombohedral, spherical, hexagonal, and cubic-like	Antioxidant	[96]
*A. indica*	Average 39.34 nm 43.63 nm	53.39 nm TEM 65.13 nm	Spherical and irregular	UV protection Antibacterial Water repellent	[113]
*D. ambrosioides*	-	Average 7, 14, 22, 70 nm FESEM	Quasi-spherical and hexagonal prisms	Antibacterial	[112]
*S. oleoides*	-	26.62 and 38.62 nm TEM	Irregular, spherical and round	Antibacterial	[104]

## Data Availability

All of the relevant data are available from the corresponding authors upon reasonable request. Source data are provided in this paper.
